# Development of a preliminary conceptual model of the patient experience of chronic kidney disease: a targeted literature review and analysis

**DOI:** 10.1186/s12882-021-02440-9

**Published:** 2021-06-23

**Authors:** Jennifer E. Flythe, Niklas Karlsson, Anna Sundgren, Paul Cordero, Amanda Grandinetti, Henry Cremisi, Anna Rydén

**Affiliations:** 1 Division of Nephrology and Hypertension, Department of Medicine, University of North Carolina School of Medicine, 7024 Burnett-Womack CB #7155, Chapel Hill, NC USA; 2 R&D Digital Health, AstraZeneca, Gothenburg, Sweden; 3 Cardiovascular Renal & Metabolic late stage development, AstraZeneca, Gaithersburg, MD USA; 4 IQVIA, Reading, UK; 5 Northern Illinois University, DeKalb, IL USA; 6 US Medical Affairs, Renal, AstraZeneca, Wilmington, DE USA

**Keywords:** Chronic kidney disease (CKD), Conceptual model, Health-related quality of life (HRQOL), Patient experience, Patient-reported outcome (PRO)

## Abstract

**Background:**

Patient-reported outcome (PRO) instruments should capture the experiences of disease and treatment that patients consider most important in order to inform patient-centred care and product development. The aim of this study was to develop a preliminary conceptual model of patient experience in chronic kidney disease (CKD) based on a targeted literature review and to characterize existing PRO instruments used in CKD.

**Methods:**

PubMed, EMBASE and Cochrane databases and recent society meetings were searched for publications reporting signs/symptoms and life impacts of CKD. Concepts identified in the literature review were used to develop a preliminary conceptual model of patient experience of CKD, overall, and within patient subpopulations of differing CKD causes, severities and complications. PRO instruments, identified from PRO databases, CKD literature and CKD clinical trials, were assessed for content validity, psychometric strength and coverage of concepts in the literature review.

**Results:**

In total, 100 publications met criteria for analysis; 56 signs/symptoms and 37 life impacts of CKD were identified from these sources. The most frequently mentioned signs/symptoms were pain/discomfort (57% of publications) and tiredness/low energy/lethargy/fatigue (42%); the most commonly reported life impacts were anxiety/depression (49%) and decrements in physical functioning (43%). Signs/symptoms and life impacts varied across the subpopulations and were more frequent at advanced CKD stages. The preliminary conceptual model grouped signs/symptoms into seven domains (pain/discomfort; energy/fatigue; sleep-related; gastrointestinal-related; urinary-related; skin−/hair−/nails-related; and other) and life impacts into six domains (psychological/emotional strain; cognitive impairment; dietary habit disruption; physical function decrements; interference with social relationships; and other). Eleven PRO instruments were considered to be promising for use in CKD; all had limitations.

**Conclusions:**

Although preliminary, the proposed conceptual model highlights key PROs for people with CKD and is intended to spur development of more tailored PRO instruments to assess these concepts.

**Supplementary Information:**

The online version contains supplementary material available at 10.1186/s12882-021-02440-9.

## Background

Chronic kidney disease (CKD), defined as an estimated glomerular filtration rate (eGFR) of less than 60 mL/min per 1.73m^2^ and/or markers of kidney damage that have been present for more than 3 months [1], is a serious global health issue. The prevalence of CKD is high, affecting an estimated 9.1% of the world’s population (approximately 700 million people) in 2017 [2], and the health impacts of CKD are substantial in terms of both mortality and morbidity [3]. The ranking of CKD among leading causes of death has continued to rise, in parallel with an increasing burden of risk factors including diabetes, hypertension and obesity; in 2017, there were an estimated 1.2 million deaths from CKD worldwide, and CKD was ranked as the twelfth leading cause of death, compared with the seventeenth leading cause of death in 1990 [2]. Moreover, CKD has a considerable impact on patient functioning and health-related quality of life (HRQOL) [3], and such impairments worsen as kidney function declines [4–9].

In recent years, there has been increasing recognition of the importance of capturing the patient experience of disease and treatment in both regulatory and clinical domains [10–13]. Better understanding of patients’ experiences can facilitate dialogue between patients and clinicians and inform treatment decisions, as well as provide important information for regulators, payers, clinicians and drug developers, supplementary to traditional efficacy and safety data. Patient-reported outcome (PRO) instruments serve this function; however, to be meaningful, such instruments must accurately capture concepts that are both relevant and important to their target population [12, 14, 15].

A wide range of generic and disease-specific PRO instruments have been used in CKD [16–18]. However, there is lack of consensus regarding the optimal PRO instruments to capture CKD patient experiences [18, 19]. Furthermore, it is plausible that CKD subpopulations such as individuals with differing disease causes, severities and complications may have different experiences and thus require nuanced PRO instruments [20–23]. To-date, there has been less focus on PRO instruments in such CKD subpopulations.

The aims of this study were to identify the most common patient-reported signs/symptoms and life impacts of CKD through a targeted literature review and to use the findings from the literature review to develop a preliminary conceptual model of the signs/symptoms and life impacts of CKD in the overall CKD population and in select subpopulations. We also sought to identify and characterize PRO instruments previously used to measure CKD patient experiences and to map them to the signs/symptoms and life impacts identified in the literature analysis.

## Methods

### Literature searches and screening

A comprehensive literature search was conducted in the PubMed database to identify articles reporting signs/symptoms of CKD and their impact on HRQOL. The search strategy combined terms related to disease, outcome and study design to identify potentially relevant references. Search terms for disease included “chronic kidney disease”, “end-stage renal disease”, “microalbuminuria”, “macroalbuminuria” and “dialysis”, while those for outcome included “quality of life”, “patient reported outcome measures” and “health utility” plus a number of named PRO instruments. The terms for study design included “observational study”, “interventional study”, “RCT”, “systematic review”, “patient interview” and “patient experience” (see **Supplementary Table S****1** for full search details).

The searches were conducted on 6 November 2019 and were restricted to English language publications and to articles published in the last 10 years. Additional literature searches were conducted in the EMBASE and Cochrane databases to identify (using the same predefined criteria) potential references that were not identified in PubMed. Recent annual society meetings (2018 and 2019), including the American Society of Nephrology, the European Renal Association–European Dialysis and Transplant Association, the International Society for Pharmacoeconomics and Outcomes Research and the International Society of Nephrology, were also searched for relevant congress abstracts.

The search results were initially screened for relevance by title, then potentially relevant references were screened by abstract to select articles for full text review based on predefined criteria (see **Supplementary Table S****2** for details). For inclusion, publications had to include patients with CKD and had to report signs/symptoms and/or life impacts of CKD and/or its treatments. Interventional, observational and epidemiological studies were included, as were reviews, instrument evaluation or development studies, patient interview studies and expert guidelines. Editorials and case reports were excluded. Article screening was performed by three reviewers (P. Cordero [PC; author], N. Singhal [NS] and S. Bondugula [SB]; all IQVIA) experienced in conducting qualitative research and systematic literature searches. A sample of 100 publications was jointly assessed to ensure alignment across reviewers. Priority was given to qualitative research articles and those including patients from the subpopulations of interest (described below). The agreement of at least two reviewers was required for publications to be included in the analysis.

### Literature analysis and conceptual model development

Signs/symptoms and life impacts of CKD were extracted from the selected publications to inform the development of a preliminary conceptual model of the effects of CKD on patients and their HRQOL. The occurrence of all patient-reported signs/symptoms and life impacts in the articles was recorded, and data on prevalence and severity were collected when available.

Analyses were conducted for all patients with CKD, and then repeated in the prespecified subpopulations of CKD causes (diabetes), CKD complications (anaemia, hyperkalaemia and cardiovascular) and CKD severity (stages 1–3, 4–5 and dialysis-dependent).

Data extraction was performed manually by three reviewers (PC, NS and SB), and a sample of articles (~ 10%) was checked by one of the other reviewers; any disagreements were resolved through discussion until consensus was reached. Based on the concepts extracted from the literature, a preliminary conceptual model of the patient experience of CKD was developed. Signs/symptoms and life impacts were identified, and related concepts were grouped. Finally, findings were compared across CKD patient subpopulations.

### PRO instrument searches and screening

Four data sources were used to identify PRO instruments: ClinicalTrials.gov; ePROVIDE databases (including PROQOLID and PROLABELS); articles from the literature searches (above); and selected literature reviews and reports.

ClinicalTrials.gov was searched for phase 2, 3 or 4 studies in CKD using PRO instruments. The search was limited to trials first posted between October 2014 and October 2019, and studies that were either suspended, terminated or withdrawn were excluded. A reviewer (SB, supervised by NS) manually searched the study outcomes to identify all clinical trials that used PRO endpoints. PRO instruments were also identified from the PROQOLID database using disease-related search terms adapted from the literature searches (above). The same terms were also used to search the PROLABELS database, and a reviewer (PC) manually searched the outcomes to identify PRO instruments included in the product labelling that were considered to be potentially relevant for CKD. Additional PRO instruments were obtained from the targeted literature review as well as five key published literature reviews [16, 24–27] and one report [28].

Duplicate PRO instruments were excluded, as were instruments unrelated to CKD or any of its related signs/symptoms and life impacts, and those outside the scope of the project (e.g. for paediatric or transplant patients). The remaining candidate PRO instruments were categorized according to their content and targeted population.

The final selection of PRO instruments for analysis was a qualitative process, taking into account the frequency of appearances of PRO instruments in: psychometric-based articles in CKD (identified from PubMed); the 100 publications selected for full text analysis plus the published literature reviews [16, 24–27] and key report [28]; and the CKD trials identified from ClinicalTrials.gov. It also took into account whether or not the PRO instruments appeared in CKD-related submissions in the Health Technology Assessment (HTA) Accelerator database (an IQVIA database containing information from HTA reports from more than 26,000 payer publications in 250 diseases across 100 agencies and 40 countries).

### PRO instrument analysis

The PubMed and PROQOLID databases, together with PRO instrument and society internet resources, were searched for articles providing development history, content validity and in-depth psychometric performance information for the selected PRO instruments. Data were extracted from two or three key articles for each instrument for analysis, including content and psychometric assessments (see **Supplementary Table S****3** for full details), based on the principles set out in the Consensus-Based Standards for the Selection of Health Measurement Instruments (COSMIN) guideline for assessing PRO measures [29]. Content validity strength was categorized as strong, medium or weak based on the development process (i.e. whether the instrument was developed for kidney diseases, and whether the development process involved patients, literature reviews and clinician experts), and psychometric strength was categorized as strong, medium or weak based on the number of psychometric properties evaluated that met the predefined thresholds (Table 1 and **Supplementary Table S****3**) [30–34].

**Table 1:** Criteria used to assess content validity strength and psychometric strength

	Content validity strength	Psychometric strength
**Measures**	• Target patient indication • Development process – Literature reviews (concepts or instruments) – Expert input/interviews – Patient participation (concept elicitation and/or cognitive debriefing interviews)	• Internal consistency reliability [30] *Cronbach’s alpha > 0.7* • Reproducibility/test–retest reliability [31, 32] *ICC > 0.7* • Construct validity [33] *Correlation coefficients range from ≥ 0.3 to ≥ 0.6* • Minimum clinically important difference [34] *Value(s) reported for PRO instrument* • Ability to detect change *Demonstrated for PRO instrument*
**Scoring**		
*Strong*	• Developed for target indication • Development process with involvement of patients, literature reviews and clinician experts	• 4 or 5 psychometric properties meeting threshold values
*Medium*	• Developed for target indication • Development process lacking patient input	• 2 or 3 psychometric properties meeting threshold values
*Weak*	• Not developed for target indication OR • Just one type of input in the development process	• 0 or 1 psychometric properties meeting threshold values

Threshold values for psychometric strength are shown in italics

*ICC* intraclass correlation coefficient, *PRO* patient-reported outcome

The PRO instruments were also assessed for coverage of the signs/symptoms and life impacts of CKD identified by conceptual modelling for each of the patient subpopulations, as well as for their presence in kidney disease-related clinical trials based on the ClinicalTrials.gov search and for their presence in product labelling from the US Food and Drug Administration (FDA) and/or the European Medicines Agency (EMA) that supports labelling claims based on the PROLABELS database search.

## Results

### Literature searches and screening

**Fig. 1:**
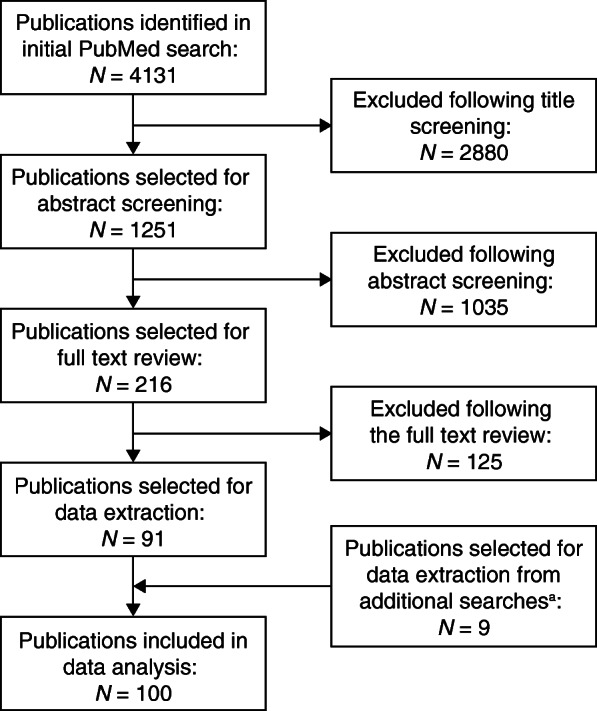
Flow diagram of screening and evaluation process to identify articles reporting patient experience in CKD. ^a^Additional searches of EMBASE and Cochrane databases, and congress abstracts. CKD, chronic kidney disease

The initial PubMed literature search identified 4131 articles for screening (Fig. 1). In total, 1251 publications were selected as being potentially relevant after an initial title screen, of which 216 were chosen for full text review after screening by abstract. Of these 216 articles, 91 were selected for data extraction. A further nine publications identified from the additional literature searches were added, yielding a total of 100 publications for analysis.

Studies focusing primarily on PRO instruments as endpoints made up just over half (57%) of the publications selected for data extraction. Qualitative studies accounted for 20% of articles, while review articles made up a further 15% of publications. Of the selected articles, 58 were published in the last 3 years of the search period (2017–2019). Twenty articles presented data from multiple countries, with the remaining 80 publications each reporting information from just one country (covering 23 countries in total), of which the USA (*n* = 20), the UK (*n* = 13), Australia (*n* = 9) and Canada (*n* = 6) were the most common. Some of the selected publications provided data for more than one patient subpopulation, so the subpopulation analyses involved a total of 115 individual patient groups. No publications were identified that focused specifically on CKD patients with hyperkalaemia or cardiovascular complications, so these subpopulations were excluded from further consideration.

### Literature analysis

For the overall CKD population, we identified 56 signs/symptoms and 37 life impacts. Similar signs/symptoms and life impacts were combined, yielding 40 signs/symptoms and 28 life impacts for analysis (see **Supplementary Tables S****4** and **S****5** for details).

The signs/symptoms most frequently reported were pain/discomfort (57%) and tiredness/low energy/lethargy/fatigue (42%). Disturbed sleep/sleep-related problems and itching/skin problems were reported in 28 and 25% of publications, respectively (Table 2). The highest reported prevalence exceeded 70% for all four signs/symptoms, although values varied widely across the publications (pain/discomfort, 10–73%; tiredness/low energy/lethargy/fatigue, 31–100%; disturbed sleep/sleep-related, 14–94%; itching/skin problems, 6–89%) (**Supplementary Table S****4**). The most commonly reported life impacts of CKD were anxiety/depression (49%), physical functioning decrement (43%), emotional distress (37%) and social interruption (34%) (Table 2). The range of prevalence values for each of these impacts was 5–83%, 14–83%, 34–82% and 5–60%, respectively (**Supplementary Table S****5**).

**Table 2:** Frequency of mentions and prevalence of signs/symptoms and life impacts in the selected publications

	Overall		Subpopulations^**a**^											
	(***N*** = 100)		General (***N*** = 48)		Diabetes (***N*** = 11)		Anaemia (***N*** = 7)		CKD 1–3 (***N*** = 9)		CKD 4–5 (***N*** = 14)		Dialysis (***N*** = 26)	
	**Freq.**	**Prev.**	**Freq.**	**Prev.**	**Freq.**	**Prev.**	**Freq.**	**Prev.**	**Freq.**	**Prev.**	**Freq.**	**Prev.**	**Freq.**	**Prev.**
Signs/symptoms^b^														
Pain/discomfort	57	**73**	58	**75**	36	NR	71	**68**	44	**60**	57	**100**	73	**75**
Tiredness/low energy/lethargy/fatigue	42	**100**	31	**100**	27	NR	86	**50**	22	24	36	**76**	62	**71**
Disturbed sleep/sleep-related problems	28	**94**	33	**94**	–	–	–	–	11	**55**	29	**66**	38	**80**
Itching/skin problems	25	**89**	17	**89**	18	NR	–	–	22	NR	29	**84**	42	**83**
Muscular pain/cramps	18	**89**	17	**89**	18	44	–	–	11	55	7	**83**	31	**69**
Appetite loss/anorexia	18	**67**	15	**67**	9	NR	–	–	–	–	21	32	35	**53**
Nausea	18	**59**	8	38	18	NR	–	–	–	–	21	**59**	38	42
Dyspnoea/shortness of breath	17	**80**	12	**67**	9	36	14	NR	–	–	21	**80**	31	**66**
Feeling unwell	7	**55**	4	NR	27	**52**	–	–	11	NR	7	NR	8	36
Dizziness	7	**50**	–	–	–	–	–	–	–	–	–	–	27	**50**
Life impacts^b^														
Anxiety/depression	49	**83**	56	39	27	**83**	14	NR	55	33	43	**65**	54	**57**
Mental impact	26	26	27	21	55	26	43	NR	–	–	7	NR	23	NR
Mood change/irritability	12	**50**	10	**50**	36	NR	–	–	11	NR	14	NR	8	24
Physical functioning	43	**83**	52	**83**	55	NR	86	NR	22	**61**	14	NR	35	14
Emotional interference	37	**82**	35	**82**	45	NR	43	NR	–	–	14	NR	54	45
Social relationship interference	34	**60**	31	**89**	64	NR	57	**83**	11	26	7	NR	38	28
Cognitive impairment (memory, concentration, confusion)	27	**61**	31	**71**	9	36	–	–	33	14	14	NR	35	**52**
General health perception	26	36	27	19	27	NR	57	NR	11	NR	7	NR	31	36
ADL/daily/regular activities	24	**80**	25	46	–	–	28	**83**	33	49	14	**80**	38	**60**
Diet/food changes/related	23	**83**	23	**74**	36	43	–	–	–	–	7	40	27	48
Vitality	23	**60**	19	24	36	NR	86	**60**	22	NR	14	NR	23	NR
Mobility problems	16	**100**	21	**61**	9	NR	14	NR	33	45	21	**100**	19	**70**
Self-care issues	10	**88**	8	NR	9	NR	28	NR	22	18	21	**68**	12	**88**
Activity impairment	9	**89**	6	**89**	18	NR	43	22	11	**52**	7	48	12	**52**

Freq. (frequency): values indicate the proportion (%) of publications in which each sign/symptom or life impact was mentioned (−, not mentioned)

Prev. (prevalence): values indicate the highest observed prevalence (%) of the signs/symptoms or life impacts in the publications (−, not mentioned). Prevalence values of 50% or above are highlighted in bold

^a^Publications could report data for more than one subpopulation

^b^Signs/symptoms and life impacts mentioned in at least 25% of the publications for any subpopulation were included

*ADL* activities of daily living, *CKD* chronic kidney disease, *freq.* frequency, *prev.* prevalence, *NR* not reported

The signs/symptoms and life impacts experienced by patients varied across the CKD subpopulations (Table 2 and **Supplementary Tables S****6** and **S****7**). Pain/discomfort was prominent across all subpopulations and was mentioned in 36–73% of publications, with prevalence values ranging from 60 to 100% across the subpopulations with available data. Mentions of pain/discomfort were more frequent among publications evaluating patients with anaemia and those with diabetic CKD (71 and 36%, respectively); no publication mentioned sleep-related problems in these two subpopulations (Table 2).

Pain/discomfort, anxiety/depression, impacts on daily or regular activities, mobility problems and cognitive impairment were reported in at least 33% of publications in patients with CKD stages 1–3 (Table 2). Analysis across CKD stages showed that the number of signs/symptoms and life impacts increased with increasing disease severity. Patients with CKD stages 1–3 experienced 12 signs/symptoms and 13 life impacts compared with 27 and 18, respectively, among patients with CKD stages 4–5, and 35 and 28, respectively, among dialysis patients (**Supplementary Tables S****6** and **S****7**). Mentions of specific signs/symptoms increased in frequency with disease severity. For example, pain/discomfort and energy/fatigue-related signs/symptoms were mentioned in 73 and 62% of publications, respectively, for the dialysis population, compared with 57 and 36%, respectively, for the CKD stages 4–5 population and 44 and 22%, respectively, for the CKD stages 1–3 population (Table 2). Similar patterns were observed for life impacts of CKD.

### Preliminary conceptual model development

**Fig. 2:**
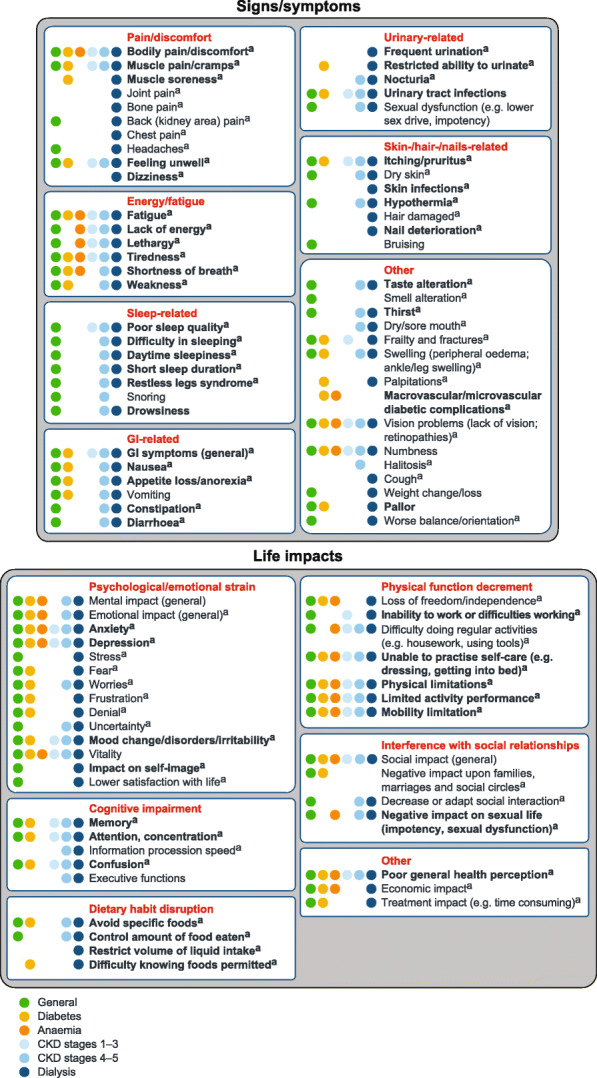
Preliminary conceptual model of patient experience in CKD based on literature review. Figure shows the signs/symptoms and life impacts identified in the targeted literature review. Presence of the concepts in each of the patient subpopulations is indicated by coloured circles. Bold represents > 50% prevalence of concept (based on highest observed prevalence in the literature) ^a^Present in qualitative literature. CKD, chronic kidney disease; GI, gastrointestinal.

Based on the literature review findings, we developed a preliminary conceptual model summarizing the signs/symptoms and life impacts experienced by patients with CKD. Figure 2 shows the signs/symptoms and life-impacts for patients with CKD, overall, and within the six subpopulations.

The signs/symptoms were grouped into seven domains: pain/discomfort, energy/fatigue, sleep-related, gastrointestinal-related, urinary-related, skin−/hair−/nails-related and other. Other signs/symptoms included frailty and fractures, swelling (oedema), vision problems and numbness. Life impacts were grouped into six domains: psychological/emotional strain, cognitive impairment, dietary habit disruption, physical function decrements, interference with social relationships and other (which included poor perception of general health and financial stress).

### Identification and analysis of PRO instruments

We identified 138 candidate PRO instruments assessing CKD experiences. Generic concept-specific instruments were the most common PRO instruments (*n* = 75; 54%), with generic HRQOL instruments accounting for an additional 39 instruments (28%). There were 24 kidney disease-specific instruments (17%), of which 17 (12% overall) assessed HRQOL and 7 (5%) evaluated specific disease concepts (**Supplementary Table S****8**).

Of the 138 candidate PRO instruments, 35 were selected for full analysis based on frequency of appearance in the CKD literature and in CKD clinical trials, and their presence in CKD-related submissions in the HTA Accelerator database. Table 3 summarizes the selected instruments and their associated psychometric properties, and content validity, and coverage of signs/symptoms and life impacts identified by the conceptual modelling.

**Table 3:** Summary of selected PRO instruments (*N* = 35), including psychometric strength, content validity and concept coverage

		Generic HRQOL				Generic concept-specific							
		SF-36		EQ-5D		BDI (BDI-II)	PHQ (PHQ-9)		HADS	TMT	HAP	RAPA^**a**^	QDIS-7^**a**^
Time to completion		5–10 min		8 min		5–10 min	10 min		2–5 min	5–10 min	5–10 min	< 2 min	< 1 min
Items		36		5 + 1 VAS (20 cm)		21	9		14	2 section	102	9	7
Recall		Past 4 wks/ past wk		Today		2 wks/ including today	Past month		Past wk	Present	7 d	NR	Past 4 wks
Languages		160		181		73	86		116	46	NA	NR	NR
Presence in trials		Yes		Yes		Yes	Yes		No	No	No	No	No
Presence in labels		Yes		Yes		Yes	No		Yes	No	No	No	No
Psychometric strength		Strong		Moderate		Weak	Weak		Moderate	Weak	Moderate	Moderate	Moderate
Population		CKD with anaemia		Pre-dialysis CKD		CKD or dialysis	Dialysis		Dialysis	Dementia	CKD-dialysis	Healthy adults	CKD
Content validity		Weak		Weak		Weak	Weak		Weak	Weak	Weak	Weak	Weak
Population		Generic		Generic		Depression	Anxiety		Anxiety/ depression	Brain injury	Lung disease	Generic	Generic
Signs/symptoms													
General		*2/30*		*1/30*		5/30	3/30		*0/30*	*0/30*	*1/30*	*1/30*	*2/30*
Diabetes		*2/19*		*1/19*		*2/19*	*2/19*		*0/19*	*0/19*	*1/19*	*1/19*	*1/19*
Anaemia		*2/6*		*1/6*		*1/6*	*1/6*		*0/6*	*0/6*	*1/6*	*0/6*	*1/6*
CKD 1–3		*2/12*		*1/12*		*2/12*	*2/12*		*0/12*	*0/12*	*0/12*	*0/12*	*2/12*
CKD 4–5		*2/26*		*1/26*		4/26	3/26		*0/26*	*0/26*	*1/26*	*1/26*	*2/26*
Dialysis		*2/31*		*1/31*		5/31	3/31		*0/31*	*0/31*	*1/31*	*1/31*	*2/31*
Life impacts													
General		**8/28**		5/28		**9/28**	**7/28**		5/28	*2/28*	*2/28*	*1/28*	**12/28**
Diabetes		**7/20**		4/20		**6/20**	**6/20**		3/20	*1/20*	*1/20*	*1/20*	**9/20**
Anaemia		**6/14**		4/14		4/14	5/14		*2/14*	*0/14*	*2/14*	*1/14*	**7/14**
CKD 1–3		**6/13**		5/13		**6/13**	5/13		*2/13*	*2/13*	*2/13*	*1/13*	**9/13**
CKD 4–5		**7/18**		5/18		**6/18**	**6/18**		3/18	*2/18*	*2/18*	*1/18*	**10/18**
Dialysis		**8/28**		5/28		**9/28**	**7/28**		5/28	*2/28*	*2/28*	*1/28*	**12/28**
**Generic concept-specific**													
	**BPI**	**FACIT-F**	**PSQI**	**SKINDEX**	**RLS-6**	**CSI**	**FACT-An**^**b**^	**HGS**	**WPAI**	**FAACT**^**b**^	**SDS (Zung)**^**a**^		
Time to completion	10 min	8–10 min	5–10 min	NR	NR	NR	10 min	NR	NR	5–10 min	10 min		
Items	56	40	24	10	6	33	47	NR	6	39	20		
Recall	Past wk	Past 7 d	Past month	NR	7 d/ nights	Past 6 months	Past 7 d	NR	Current/ past 7 d	Past 7 d	In general		
Languages	52	58	77	NR	NR	NA	53	NR	70	31	NR		
Presence in trials	No	Yes	Yes	Yes	Yes	No	Yes	No	No	Yes	No		
Presence in labels	Yes	Yes	Yes	Yes	No	No	Yes	Yes	Yes	No	No		
Psychometric strength	Moderate	Moderate	Moderate	Weak	Moderate	Weak	Strong	Moderate	Weak	Weak	Weak		
Population	CKD-dialysis	ESRD-dialysis	Renal transplant	Skin diseases	RLS	Dialysis	CKD with anaemia	CKD-dialysis	Anaemia	Cancer/HIV with anorexia	Depression		
Content validity	Weak	Weak	Weak	Weak	Weak	Weak	Weak	Weak	Weak	Weak	Weak		
Population	Pain	Chronic disease	Sleep disorder	Pruritus	RLS	Generic	Anaemia	Generic	Anaemia	Anorexia/ cachexia	Depression		
Signs/symptoms													
General	3/30	*2/30*	5/30	*1/30*	3/30	*0/30*	**7/30**	*1/30*	*0/30*	**6/30**	5/30		
Diabetes	*2/19*	*1/19*	*2/19*	*1/19*	*1/19*	*0/19*	4/19	*1/19*	*0/19*	5/19	*1/19*		
Anaemia	*1/6*	*1/6*	*2/6*	*0/6*	*1/6*	*0/6*	3/6	*0/6*	*0/6*	*2/6*	*1/6*		
CKD 1–3	*2/12*	*2/12*	*2/12*	*1/12*	*2/12*	*0/12*	3/12	*0/12*	*0/12*	*2/12*	*2/12*		
CKD 4–5	*2/26*	*2/26*	4/26	*1/26*	3/26	*0/26*	**6/26**	*1/26*	*0/26*	**6/26**	5/26		
Dialysis	3/31	*2/31*	4/31	*1/31*	3/31	*0/31*	**8/31**	*1/31*	*0/31*	**7/31**	5/31		
Life impacts													
General	**6/28**	3/28	3/28	**7/28**	**6/28**	*2/28*	**9/28**	*0/28*	*1/28*	**10/28**	4/28		
Diabetes	4/20	*2/20*	*2/20*	4/20	4/20	*2/20*	**6/20**	*0/20*	*0/20*	**7/20**	3/20		
Anaemia	3/14	*2/14*	*2/14*	*2/14*	4/14	*1/14*	**6/14**	*0/14*	*0/14*	**6/14**	3/14		
CKD 1–3	5/13	*2/13*	*2/13*	4/13	5/13	*2/13*	5/13	*0/13*	*1/13*	4/13	*2/13*		
CKD 4–5	3/18	*2/18*	*2/18*	3/18	5/18	*2/18*	**7/18**	*0/18*	*0/18*	**8/18**	3/18		
Dialysis	**6/28**	3/28	3/28	**7/28**	**6/28**	*2/28*	**9/28**	*0/28*	*1/28*	**10/28**	4/28		
**Kidney disease-specific HRQOL**													
	**KDQOL-36**	**KDQOL-SF**	**KDQOL**	**CHEQ**	**DSI**	**WHOQOL-BREF Dial.**	**POS-S Renal**	**QLI-D**	**CKD QOL**	**KDBI**	**CKD-SBI**		
Time to completion	NR	16 min	30 min	NR	NR	5/15–20 min	NR	5–10 min	NR	NR	Approx. 10 min		
Items	36	80	134	86	30	26	18	68	34	16	25		
Recall	1 d/past 4 wks	Past 4 wks	Past 4 wks	4 wks	Past 7 d	Past 2–4 wks	1 wk	NR	Past 4 wks	Past month	7 d		
Languages	37	25	41	2	NR	51	NR	6	NR	NR	1		
Presence in trials	Yes	Yes	Yes	No	Yes	No	Yes	No	No	No	No		
Presence in labels	No	No	No	No	No	No	No	No	No	No	No		
Psychometric strength	Moderate	Weak	Weak	Weak	Moderate	Moderate	Moderate	Moderate	Moderate	Moderate	Moderate		
Population	CKD	Kidney disease-dialysis	Kidney disease-dialysis	ESRD-dialysis	CKD- dialysis	CKD- dialysis	Advanced CKD	ESRD- dialysis	CKD (3–5, dialysis & transplant)	ESRD	CKD		
Content validity	Moderate	Moderate	Moderate	Strong	Strong	Strong	Moderate	Strong	Strong	Moderate	Strong		
Population	Kidney disease	Kidney disease-dialysis	Kidney disease-dialysis	Kidney disease-dialysis	Dialysis	CKD (4–5)	CKD (4–5)	Kidney disease-dialysis	CKD (3–5, dialysis & transplant)	Kidney disease	CKD		
Signs/symptoms													
General	**9/30**	**9/30**	3/30	3/30	**16/30**	*0/30*	**14/30**	*1/30*	*2/30*	*0/30*	**13/30**		
Diabetes	**8/19**	**8/19**	*2/19*	*1/19*	**9/19**	*0/19*	**9/19**	*1/19*	*2/19*	*0/19*	**8/19**		
Anaemia	4/6	4/6	*2/6*	*1/6*	**6/6**	*0/6*	3/6	*1/6*	*2/6*	*0/6*	3/6		
CKD 1–3	**6/12**	**6/12**	*2/12*	*2/12*	**6/12**	*0/12*	**6/12**	*1/12*	*2/12*	*0/12*	4/12		
CKD 4–5	**9/26**	**9/26**	3/26	3/26	**15/26**	*0/26*	**14/26**	*1/26*	*2/26*	*0/26*	**12/26**		
Dialysis	**10/31**	**10/31**	3/31	3/31	**17/31**	*0/31*	**15/31**	*1/31*	*2/31*	*0/31*	**13/31**		
Life impacts													
General	**13/28**	**13/28**	**12/28**	**14/28**	5/28	5/28	*2/28*	4/28	5/28	4/28	*2/28*		
Diabetes	**10/20**	**10/20**	**9/20**	**12/20**	4/20	4/20	*2/20*	3/20	5/20	*2/20*	*2/20*		
Anaemia	**9/14**	**9/14**	**10/14**	**11/14**	*2/14*	5/14	*2/14*	4/14	4/14	*1/14*	*0/14*		
CKD 1–3	**8/13**	**8/13**	**8/13**	**8/13**	*2/13*	4/13	*2/13*	3/13	4/13	*1/13*	*2/13*		
CKD 4–5	**10/18**	**10/18**	**10/18**	**11/18**	5/18	4/18	*2/18*	4/18	5/18	*2/18*	*2/18*		
Dialysis	**13/28**	**13/28**	**12/28**	**14/28**	5/28	5/28	*2/28*	4/28	5/28	4/28	*2/28*		
**Kidney disease-specific**													
		**ESRD-SI**		**HSS**			**FAS**	**FSGS**					
Time to completion		NR		NR			NR	NR					
Items		NR		29			10	17					
Recall		NR		NR			Usually	Past 7 d					
Languages		NR		NR			2	NR					
Presence in trials		No		No			No	No					
Presence in labels		No		No			No	No					
Psychometric strength		Moderate		Moderate			Weak	Weak					
Population		ESRD- dialysis		ESRD- dialysis			ESRD- dialysis	NR					
Content validity		Moderate		Moderate			Weak	Moderate					
Population		CKD-dialysis		CKD-dialysis			Fatigue	FSGS					
Signs/symptoms													
General		**6/30**		**7/30**			*1/30*	**7/30**					
Diabetes		**7/19**		3/19			*1/19*	4/19					
Anaemia		5/6		*2/6*			*1/6*	3/6					
CKD 1–3		5/12		**6/12**			*1/12*	4/12					
CKD 4–5		**6/26**		**7/26**			*1/26*	5/26					
Dialysis		**6/31**		**7/31**			*1/31*	**7/31**					
Life impacts													
General		*0/28*		**12/28**			3/28	**13/28**					
Diabetes		*0/20*		**7/20**			3/20	**12/20**					
Anaemia		*0/14*		**8/14**			*2/14*	**9/14**					
CKD 1–3		*0/13*		**7/13**			*2/13*	**8/13**					
CKD 4–5		*0/18*		**7/18**			3/18	**11/18**					
Dialysis		*0/28*		**12/28**			3/28	**13/28**					

The coverage of signs/symptoms and life impacts by each PRO was categorized as good (≥6 concepts [in bold]), moderate (3–5) or poor (0–2 [in italics])

^a^Selected for analysis because of its potential for use in patients with CKD, although it was not among the PRO instruments identified from the data sources

^b^Instrument includes the FACT-G module

*BPI* Brief Pain Inventory, *CHEQ* CHOICE Health Experience Questionnaire, *CKD* chronic kidney disease, *CKD QOL* Chronic Kidney Disease Quality of Life, *CKD-SBI* Chronic Kidney Disease-Symptom Burden Index, *CSI* Coping Strategy Indicator, *d* days, *DSI* Dialysis Symptom Index, *EQ-5D* European quality of life – five dimension, *ESRD* end-stage renal disease, *ESRD-SI* End-Stage Renal Disease Severity Index, *FAACT* Functional Assessment of Anorexia/Cachexia Therapy, *FACIT-F* Functional Assessment of Chronic Illness Therapy-Fatigue Scale, *FACT-An* Functional Assessment of Cancer Therapy-Anemia, *FACT-G* Functional Assessment of Cancer Therapy-General, *FAS* Hemodialysis Fatigue Scale, *FSGS* Focal Segmental Glomerulosclerosis Symptom Impact Questionnaire, *HADS* Hospital Anxiety and Depression Scale, *HAP* Human Activity Profile, *HGS* hand grip strength test, *HIV* human immunodeficiency virus, *HRQOL* health-related quality of life, *HSS* Hemodialysis Stressor Scale, *KDBI* Kidney Disease Behavior Inventory, *KDQOL* Kidney Disease Quality of Life, *KDQOL-36* Kidney Disease Quality of Life-36, *KDQOL-SF* Kidney Disease Quality of Life Short Form, *NA* not applicable, *NR* not reported, *PHQ* Patient Health Questionnaire, *POS-S Renal* Palliative Care Outcome Scale-Symptoms (Renal), *PRO* patient-reported outcome, *PSQI* Pittsburgh Sleep Quality Index, *QDIS-7* Quality of Life Disease Impact Scale-7, *QLI-D* Quality of Life Index Dialysis Version, *RAPA* Rapid Assessment Physical Activity, *RLS* restless legs syndrome, *RLS-6* Restless Legs Syndrome-6 Scale, *SDS (Zung)* the Zung self-rating depression scale, *SF-36* 36-Item Short-Form Survey, *SKINDEX* quality-of-life measure for patients with skin disease, *TMT* Trail Making Test, *VAS* Visual Analogue Scale, *WHOQOL-BREF Dial.* World Health Organization Quality of Life Brief Scale in Dialysis, *wk.* week, *WPAI* Work Productivity and Activity Impairment

Analysis of the generic HRQOL instruments showed that the 36-Item Short-Form Survey (SF-36) provided good coverage of the life impacts of CKD across all subpopulations, although coverage of signs/symptoms was limited. Psychometric strength was strong for the SF-36, although evidence of content validity and psychometric validation in the CKD population was limited. The generic concept-specific instruments generally showed either poor or moderate coverage of the signs/symptoms, with only two instruments (Functional Assessment of Cancer Therapy-Anemia [FACT-An] and Functional Assessment of Anorexia/Cachexia Therapy [FAACT]) providing good coverage for any of the CKD subpopulations (Table 3). Over half of these PRO instruments (*n* = 12/20; 60%) also showed only poor or moderate coverage of the life impacts. The kidney disease-specific HRQOL instruments all showed moderate or strong content validity, which was consistent with their development for the target population, while psychometric strength was moderate to weak (Table 3). Coverage of signs/symptoms and life impacts varied widely across the instruments.

Qualitative assessments identified 11 PRO instruments as promising for use in patients with CKD based on content validity and psychometric strength, coverage of signs/symptoms and life impacts, and presence in kidney disease-specific clinical trials and FDA and EMA product labelling. The strengths and weaknesses of these instruments are summarized in Table 4. Three generic concept-specific instruments were considered to have potential for use in CKD (Table 4). FACT-An showed strong psychometric strength, with validation in the CKD-anaemia population, although additional content validation is needed. In addition, it omits such signs/symptoms as numbness, neuropathy and vision-related problems, all common in the CKD-anaemia subpopulation. FAACT showed generally good coverage of the signs/symptoms and life impacts, although this was mainly from the Functional Assessment of Cancer Therapy-General (FACT-G) instrument module, which covers aspects of patient well-being. The Beck Depression Inventory-II (BDI-II) showed good coverage of the relevant specific concepts (i.e. depression), although it had limited content and psychometric validity in CKD.

**Table 4:** Promising PROs for patients with CKD: strengths and weaknesses

PRO	Type/coverage of PRO	Strengths^**a**^	Weaknesses/gaps^**a**^
SF-36 [35–37]	Generic HRQOL	• Coverage of life impacts high • Presence in trials and labels	• Content and psychometric validity
FACT-An [38–40]	Generic concept- specific	• Psychometric validity (CKD-anaemia) • Presence in trials and labels	• Coverage of concepts mainly from FACT-G section • Content validity
FAACT [41, 42]	Generic concept- specific	• Presence in trials	• Coverage of concepts mainly from FACT-G section • No presence in labels
BDI-II [43–45]	Generic concept-specific	• Coverage of relevant specific concepts; depression • Presence in trials and labels	• Content and psychometric validity
KDQOL-36 [46–48]	Kidney disease-specific HRQOL	• Coverage of CKD-specific concepts high • Presence in trials • Content and psychometric validity (moderate)	• No presence in labels • Some psychometric validation and content validity data needed
KDQOL [49]	Kidney disease-specific HRQOL	• Presence in trials • Coverage of life impacts high	• Coverage of signs and symptoms low (for 134-item version) • Content and psychometric validity • No presence in labels
DSI [50–52]	Kidney disease-specific HRQOL	• Content and psychometric validity (moderate) • Coverage of CKD-specific signs and symptoms high • Presence in trials	• Coverage of CKD-specific life impacts low • No presence in labels • Some psychometric validation needed
POS-S Renal [53, 54]	Kidney disease-specific HRQOL	• Content and psychometric validity (moderate) • Coverage of CKD-specific concepts high • Presence in trials	• No presence in labels • Some psychometric validation needed • As a check list, it might be questionable from a regulatory perspective
CKD-SBI [55, 56]	Kidney disease-specific HRQOL	• Content and psychometric validity (moderate) • Coverage of CKD-specific signs and symptoms high	• Coverage of CKD-specific signs and symptoms low • No presence in trials and labels • Some psychometric validation needed
ESRD-SI [57, 58]	Kidney disease-specific concept-specific	• Content and psychometric validity (moderate) • Coverage of CKD-specific signs and symptoms high	• No presence in trials and labels • Coverage of CKD-specific life impacts low • Lack of information found
HSS [59, 60]	Kidney disease-specific concept-specific	• Content and psychometric validity (moderate) • Coverage of CKD-specific concepts high	• No presence in trials and labels • Lack of information found

^a^Described presence in labels is not CKD-specific

*BDI-II* Beck Depression Inventory-II, *CKD* chronic kidney disease, *CKD-SBI* Chronic Kidney Disease-Symptom Burden Index, *DSI* Dialysis Symptom Index, *ESRD-SI* End-Stage Renal Disease Severity Index, *FAACT* Functional Assessment of Anorexia/Cachexia Therapy, *FACT-An* Functional Assessment of Cancer Therapy-Anemia, *FACT-G* Functional Assessment of Cancer Therapy-General, *HRQOL* health-related quality of life, *HSS* Hemodialysis Stressor Scale, *KDQOL* Kidney Disease Quality of Life, *KDQOL-36* Kidney Disease Quality of Life-36, *POS-S Renal* Palliative Care Outcome Scale-Symptoms (Renal), *PRO* patient-reported outcomes, *SF-36* 36-Item Short-Form Survey

Among the five kidney disease-specific HRQOL instruments considered promising, the Kidney Disease Quality of Life-36 (KDQOL-36) questionnaire showed strong concept coverage for signs/symptoms and life impacts, although evidence supporting meaningful change thresholds was limited, particularly across subpopulations (Table 4). The Dialysis Symptom Index (DSI) had strong content validity and good coverage of signs/symptoms, but coverage of life impacts was low. The Palliative Care Outcome Scale-Symptoms (Renal) (POS-S Renal) and Chronic Kidney Disease-Symptom Burden Index (CKD-SBI) both had good coverage of signs/symptoms and moderate or strong content validity and psychometric strength, but coverage of life impacts was limited, and both need further psychometric validation.

The End-Stage Renal Disease Severity Index (ESRD-SI) and the Hemodialysis Stressor Scale (HSS) were the most promising kidney disease-specific PRO instruments, with both instruments demonstrating moderate psychometric strength and content validity (Table 4). Both instruments showed good or moderate coverage of signs/symptoms. Coverage of life impacts was good with HSS, but life impacts were not covered by the ESRD-SI. However, information for both instruments is limited, and further evidence supporting clinically meaningful score change is needed.

## Discussion

This literature review and analysis shows that patients with CKD experience a wide range of signs/symptoms and life impacts; 93 different signs/symptoms and life impacts were identified from the selected publications. Pain/discomfort and low energy/fatigue were the most commonly reported signs/symptoms, and anxiety/depression and decrements in physical functioning were the most frequently mentioned life impacts of CKD across all publications. Prevalence (based on highest observed value in the literature; Table 2 and **Supplementary Table S****4**) exceeded 70% for each of these signs/symptoms and life impacts.

Variations in the signs/symptoms and life impacts of CKD were observed across the six patient subpopulations, although pain/discomfort and anxiety/depression were prominent across most of them, both in terms of frequency of mentions and prevalence. In addition, the number of signs/symptoms and life impacts increased with increasing disease severity, and the frequency of mentions for signs/symptoms typically increased with disease progression. Although this literature analysis suggests that patients in early stages of CKD (stages 1–3) may already be experiencing the signs/symptoms and life impacts of the disease, this finding should be interpreted with caution. In early-stage disease, it may be difficult to determine whether such signs/symptoms can be attributed to CKD or to other comorbid conditions or factors because the biological basis for signs/symptoms is not understood as well in early disease as in more advanced disease.

Based on the literature analysis, a preliminary conceptual model was developed of patient experience of the signs/symptoms and life impacts of CKD. Signs/symptoms were grouped into seven domains related to pain/discomfort, energy/fatigue, sleep-related, gastrointestinal-related, urinary-related, skin−/hair−/nails-related and other. The life impacts fell into six domains: psychological/emotional strain, cognitive impairment, dietary habit disruption, physical function decrements, interference with social relationships and other. Analyses revealed differences in concepts across the prespecified subpopulations. For example, only pain/discomfort, energy/fatigue and “other” signs/symptoms were observed in the anaemia subpopulation. However, these findings may have been influenced by the limited number of publications available for some subpopulations. Understanding what experiences are most common in patients with CKD is an important guide for clinical research and real-world patient care practices. Our preliminary conceptual model of patient experiences in CKD is based on the existing literature, including patient interview studies. An important future step is to refine the conceptual model based on direct input from patients and clinical experts [13].

PRO instruments are important for capturing patient experience of disease and treatments, the value of which has increasingly been recognized in the drug development process [12, 13, 61]. To provide meaningful data, however, PRO instruments have to be readily understood by patients and assess the disease concepts they consider to be most important [12, 14, 15]. Among the 35 PRO instruments evaluated in this analysis, many provided only limited coverage of the signs/symptoms and life impacts for CKD identified from the literature, even among kidney disease-specific instruments, suggesting that additional population-specific work is required. Furthermore, psychometric strength was typically moderate or weak, and only two of the PRO instruments were rated as strong in CKD. This emphasizes the need for further investigations of psychometric strength for most CKD PRO instruments.

Based on our analyses, we considered 11 PRO instruments promising for use in CKD, although all have limitations or evidence gaps. Many of the PRO instruments showed limited psychometric validation in CKD, and the generic PRO instruments assessing HRQOL or concept-specific outcomes showed limited content validity in CKD. Although the generic HRQOL assessment questionnaire SF-36 showed good coverage of life impacts, its value in the clinical trial setting may be limited because other influencing, intervening factors may also affect these HRQOL outcomes over time. Disease-specific HRQOL measures, such as KDQOL-36 (which showed good concept coverage), also face similar challenges to the generic HRQOL measures because outcomes that are more proximal to the disease or treatment, such as symptoms, are more likely to show a meaningful treatment effect than downstream consequences, such as HRQOL, which are influenced by a range of factors. Given observed limitations, a potential next step would be to use the results of the preliminary conceptual modelling and PRO instrument analysis to inform the adaption of existing instruments or the development of new instruments to more comprehensively and accurately capture the patient experience of CKD for use in clinical trials and, potentially, patient care.

### Study limitations

Although the literature review was carefully designed and conducted, it does have limitations. First, there was limited relevant literature on patient experiences in some of the prespecified CKD subpopulations (i.e. dyskalaemia and cardiovascular complications). This highlights the need for focused qualitative research and conceptual modelling in these subpopulations. Second, prevalence data for CKD signs/symptoms and life impacts were limited, driven by the inconsistent inclusion of such concepts in PRO instruments. Third, there may be overlap between some concepts identified in the literature, for example between anxiety/depression, mental impact and mood change/irritability. Fourth, the review was not fully systematic; data were analysed for a subset of 100 articles selected from the relevant publications identified by the screening process. This could potentially have led to an incomplete picture of CKD-related signs/symptoms and life impacts. However, articles were selected based on prespecified criteria, and publications covering the CKD subpopulations of interest were prioritized.

## Conclusions

This literature review demonstrated the wide range of signs/symptoms and life impacts experienced by patients with CKD, of which pain/discomfort, energy/fatigue, anxiety/depression and decrements in physical functioning were the most prominent. We proposed a preliminary conceptual model of patient experience in CKD, grouping related signs/symptoms and life impacts into seven and six domains, respectively. Analysis of PRO instruments, which included coverage of the signs/symptoms and life impacts, showed limited coverage of the CKD experiences identified from the literature, even among kidney disease-specific instruments. Of the 11 promising PRO instruments identified for use in CKD, all had limitations. Direct patient input is an important next step for refining our conceptual model of patient experience in CKD and assessing PRO instruments that capture its constructs. Although preliminary, the proposed conceptual model highlights key PROs for patients with CKD and is intended to spur development of more tailored PRO instruments to assess these concepts across the entire CKD population as well as key subpopulations.

## Supplementary Information

**Additional file 1: Table S1** Search terms used for the initial PubMed literature search. **Table S2** Predefined inclusion and exclusion criteria for selecting publications for review. **Table S3** PRO instrument analysis. **Table S4** Signs/symptoms: mentions and prevalence for all publications (*N*=100). **Table S5** Life impacts: mentions and prevalence for all publications (*N* = 100). **Table S6** Signs/symptoms: mentions and prevalence by subpopulation. **Table S7** Life impacts: mention and prevalence by subpopulation. **Table S8** List of the 138 candidate PRO instruments screened for inclusion in the full analysis Generic HRQOL

## Data Availability

Data for this review were identified by searching PubMed, EMBASE and Cochrane databases and recent society meetings. The included publications are cited in the manuscript and included in the reference list.

## Abbreviations

BDI-II: Beck Depression Inventory-II
CKD: Chronic kidney disease
CKD-SBI: Chronic Kidney Disease-Symptom Burden Index
COSMIN: Consensus-Based Standards for the Selection of Health Measurement Instruments
DSI: Dialysis Symptom Index
EMA: European Medicines Agency
ESRD-SI: End-Stage Renal Disease Severity Index
FAACT: Functional Assessment of Anorexia/Cachexia Therapy
FACT-An: Functional Assessment of Cancer Therapy-Anemia
FACT-G: Functional Assessment of Cancer Therapy-General
FDA: US Food and Drug Administration
GFR: Glomerular filtration rate
HRQOL: Health-related quality of life
HSS: Hemodialysis Stressor Scale
HTA: Health Technology Assessment
KDQOL: Kidney Disease Quality of Life
KDQOL-36: Kidney Disease Quality of Life-36
POS-S Renal: Palliative Care Outcome Scale-Symptoms (Renal)
PRO: Patient-reported outcome
RCT: Randomised controlled trial
SF-36: 36-Item Short-Form Survey

## References

[1] Kidney Disease (2013). Improving Global Outcomes (KDIGO) CKD Work Group. KDIGO 2012 clinical practice guideline for the evaluation and management of chronic kidney disease. Kidney Int Suppl.

[2] GBD Chronic Kidney Disease Collaboration (2020). Global, regional, and national burden of chronic kidney disease, 1990-2017: a systematic analysis for the global burden of Disease study 2017. Lancet..

[3] Webster AC, Nagler EV, Morton RL, Masson P (2017). Chronic kidney disease. Lancet..

[4] Kittiskulnam P, Sheshadri A, Johansen KL (2016). Consequences of CKD on functioning. Semin Nephrol.

[5] Etgen T, Chonchol M, Forstl H, Sander D (2012). Chronic kidney disease and cognitive impairment: a systematic review and meta-analysis. Am J Nephrol.

[6] Weiner DE, Seliger SL (2014). Cognitive and physical function in chronic kidney disease. Curr Opin Nephrol Hypertens.

[7] Mujais SK, Story K, Brouillette J, Takano T, Soroka S, Franek C, Mendelssohn D, Finkelstein FO (2009). Health-related quality of life in CKD patients: correlates and evolution over time. Clin J Am Soc Nephrol.

[8] Pagels AA, Soderkvist BK, Medin C, Hylander B, Heiwe S (2012). Health-related quality of life in different stages of chronic kidney disease and at initiation of dialysis treatment. Health Qual Life Outcomes.

[9] Nguyen NTQ, Cockwell P, Maxwell AP, Griffin M, O'Brien T, O'Neill C (2018). Chronic kidney disease, health-related quality of life and their associated economic burden among a nationally representative sample of community dwelling adults in England. PLoS One.

[10] Reaney M, Bush E, New M, Paty J, Roborel de Climens A, Skovlund SE (2019). the potential role of individual-level benefit-risk assessment in treatment decision making: a DIA study endpoints community workstream. Ther Innov Regul Sci.

[11] Jackson Y, Janssen E, Fischer R, Beaverson K, Loftus J, Betteridge K, Rhoten S, Flood E, Lundie M (2019). The evolving role of patient preference studies in health-care decision-making, from clinical drug development to clinical care management. Expert Rev Pharmacoecon Outcomes Res.

[12] US Food and Drug Administration. Patient-reported outcome measures: use in medical product development to support labeling claims. December 2009. https://www.fda.gov/media/77832/download. Accessed 22 July 2020.

[13] US Food and Drug Administration. Patient-focused drug development: collecting comprehensive and representative input. Guidance for industry, Food and Drug Administration staff, and other stakeholders. June 2020. https://www.fda.gov/media/139088/download. Accessed 22 July 2020.

[14] Patrick DL, Burke LB, Gwaltney CJ, Leidy NK, Martin ML, Molsen E, Ring L (2011). Content validity–establishing and reporting the evidence in newly developed patient-reported outcomes (PRO) instruments for medical product evaluation: ISPOR PRO good research practices task force report: part 1–eliciting concepts for a new PRO instrument. Value Health.

[15] Patrick DL, Burke LB, Gwaltney CJ, Leidy NK, Martin ML, Molsen E, Ring L (2011). Content validity–establishing and reporting the evidence in newly developed patient-reported outcomes (PRO) instruments for medical product evaluation: ISPOR PRO good research practices task force report: part 2–assessing respondent understanding. Value Health.

[16] Nair D, Wilson FP (2019). Patient-reported outcome measures for adults with kidney disease: current measures, ongoing initiatives, and future opportunities for incorporation into patient-centered kidney care. Am J Kidney Dis.

[17] van der Willik EM, Meuleman Y, Prantl K, van Rijn G, Bos WJW, van Ittersum FJ, Bart HAJ, Hemmelder MH, Dekker FW (2019). Patient-reported outcome measures: selection of a valid questionnaire for routine symptom assessment in patients with advanced chronic kidney disease - a four-phase mixed methods study. BMC Nephrol.

[18] Verberne WR, Das-Gupta Z, Allegretti AS, Bart HAJ, van Biesen W, Garcia-Garcia G (2019). Development of an international standard set of value-based outcome measures for patients with chronic kidney disease: a report of the international consortium for health outcomes measurement (ICHOM) CKD working group. Am J Kidney Dis.

[19] Breckenridge K, Bekker HL, Gibbons E, van der Veer SN, Abbott D, Briancon S (2015). How to routinely collect data on patient-reported outcome and experience measures in renal registries in Europe: an expert consensus meeting. Nephrol Dial Transplant.

[20] Clemens KK, O'Regan N, Rhee JJ (2019). Diabetes management in older adults with chronic kidney disease. Curr Diab Rep.

[21] Winocour PH (2018). Diabetes and chronic kidney disease: an increasingly common multi-morbid disease in need of a paradigm shift in care. Diabet Med.

[22] van Nooten FE, Green J, Brown R, Finkelstein FO, Wish J (2010). Burden of illness for patients with non-dialysis chronic kidney disease and anemia in the United States: review of the literature. J Med Econ.

[23] O’Mara NB (2008). Anemia in chronic kidney disease. Diab Spectr.

[24] Danquah FV, Wasserman J, Meininger J, Bergstrom N (2010). Quality of life measures for patients on hemodialysis: a review of psychometric properties. Nephrol Nurs J.

[25] Flythe JE, Powell JD, Poulton CJ, Westreich KD, Handler L, Reeve BB, Carey TS (2015). Patient-reported outcome instruments for physical symptoms among patients receiving maintenance dialysis: a systematic review. Am J Kidney Dis.

[26] Glover C, Banks P, Carson A, Martin CR, Duffy T (2011). Understanding and assessing the impact of end-stage renal disease on quality of life: a systematic review of the content validity of self-administered instruments used to assess health-related quality of life in end-stage renal disease. Patient..

[27] Tang E, Bansal A, Novak M, Mucsi I (2017). Patient-reported outcomes in patients with chronic kidney disease and kidney transplant-part 1. Front Med (Lausanne).

[28] Gibbons E, Fitzpatrick R. A structured review of patient-reported outcome measures for people with chronic kidney disease. Patient-Reported Outcome Measurement Group, Oxford. Report to the Department of Health and NHS Kidney Care. 2010.

[29] Prinsen CAC, Mokkink LB, Bouter LM, Alonso J, Patrick DL, de Vet HCW, Terwee CB (2018). COSMIN guideline for systematic reviews of patient-reported outcome measures. Qual Life Res.

[30] Guttman L (1945). A basis for analyzing test-retest reliability. Psychometrika..

[31] Cohen JA (1960). A coefficient of agreement for nominal scales. Educ Psychol Meas.

[32] Shrout PE, Fleiss JL (1979). Intraclass correlations: uses in assessing rater reliability. Psychol Bull.

[33] Hinkle DE, Wiersma W, Jurs SG (2003). Applied statistics for the behavioral sciences.

[34] US Food and Drug Administration. Methods to identify what is important to patients & select, develop or modify fit-for-purpose clinical outcomes assessments. Patient-focused drug development guidance public workshop. 15–16 October 2018. https://www.fda.gov/downloads/Drugs/NewsEvents/UCM620711.pdf. .

[35] Ware JE, Sherbourne CD (1992). The MOS 36-item short-form health survey (SF-36). I. Conceptual framework and item selection. Med Care.

[36] Brazier JE, Harper R, Jones NM, O'Cathain A, Thomas KJ, Usherwood T, Westlake L (1992). Validating the SF-36 health survey questionnaire: new outcome measure for primary care. BMJ..

[37] McHorney CA, Ware JE, Lu JF, Sherbourne CD (1994). The MOS 36-item short-form health survey (SF-36): III. Tests of data quality, scaling assumptions, and reliability across diverse patient groups. Med Care.

[38] Cella D (1997). The functional assessment of Cancer therapy-Anemia (FACT-an) scale: a new tool for the assessment of outcomes in cancer anemia and fatigue. Semin Hematol.

[39] Cella D, Eton DT, Lai JS, Peterman AH, Merkel DE (2002). Combining anchor and distribution-based methods to derive minimal clinically important differences on the functional assessment of Cancer therapy (FACT) anemia and fatigue scales. J Pain Symptom Manag.

[40] Finkelstein FO, van Nooten F, Wiklund I, Trundell D, Cella D (2018). Measurement properties of the short Form-36 (SF-36) and the functional assessment of Cancer therapy - Anemia (FACT-an) in patients with anemia associated with chronic kidney disease. Health Qual Life Outcomes.

[41] Ribaudo JM, Cella D, Hahn EA, Lloyd SR, Tchekmedyian NS, Von Roenn J (2000). Re-validation and shortening of the functional assessment of anorexia/Cachexia therapy (FAACT) questionnaire. Qual Life Res.

[42] Molfino A, Kaysen GA, Chertow GM, Doyle J, Delgado C, Dwyer T, Laviano A, Rossi Fanelli F, Johansen KL (2016). Validating appetite assessment tools among patients receiving hemodialysis. J Ren Nutr.

[43] Watnick S, Wang PL, Demadura T, Ganzini L (2005). Validation of 2 depression screening tools in dialysis patients. Am J Kidney Dis.

[44] Hedayati SS, Minhajuddin AT, Toto RD, Morris DW, Rush AJ (2009). Validation of depression screening scales in patients with CKD. Am J Kidney Dis.

[45] Loosman WL, Siegert CE, Korzec A, Honig A (2010). Validity of the hospital anxiety and depression scale and the Beck depression inventory for use in end-stage renal disease patients. Br J Clin Psychol.

[46] Ricardo AC, Hacker E, Lora CM, Ackerson L, DeSalvo KB, Go A, Kusek JW, Nessel L, Ojo A, Townsend RR, Xie D, Ferrans CE, Lash JP, CRIC Investigators (2013). Validation of the kidney Disease quality of life short form 36 (KDQOL-36) US Spanish and English versions in a cohort of Hispanics with chronic kidney disease. Ethn Dis.

[47] Peipert JD, Bentler PM, Klicko K, Hays RD (2018). Psychometric properties of the kidney Disease quality of life 36-item short-form survey (KDQOL-36) in the United States. Am J Kidney Dis.

[48] Mateti UV, Nagappa AN, Attur RP, Nagaraju SP, Mayya SS, Balkrishnan R (2015). Cross-cultural adaptation, validation and reliability of the South Indian (Kannada) version of the Kidney Disease and Quality of Life (KDQOL-36) instrument. Saudi J Kidney Dis Transpl.

[49] Hays RD, Kallich JD, Mapes DL, Coons SJ, Carter WB (1994). Development of the kidney disease quality of life (KDQOL) instrument. Qual Life Res.

[50] Weisbord SD, Fried LF, Arnold RM, Rotondi AJ, Fine MJ, Levenson DJ, Switzer GE (2004). Development of a symptom assessment instrument for chronic hemodialysis patients: the Dialysis symptom index. J Pain Symptom Manag.

[51] Önsöz HB, Usta YÖ (2013). Reliability and validity of the Turkish version of the Dialysis symptom index in chronic hemodialysis patients. Turk Neph Dial Transpl.

[52] Zamanian H, Taheri KZ (2015). Translation and psychometric properties of the Persian version of the dialysis symptom index in hemodialysis patients. Nephrourol Mon.

[53] Gutierrez-Sanchez D, Leiva-Santos JP, Sanchez-Hernandez R, Hernandez-Marrero D, Cuesta-Vargas AI (2016). Spanish modified version of the palliative care outcome scale-symptoms renal: cross-cultural adaptation and validation. BMC Nephrol.

[54] Raj R, Ahuja K, Frandsen M, Murtagh FEM, Jose M (2018). Validation of the IPOS-renal symptom survey in advanced kidney disease: a cross-sectional study. J Pain Symptom Manag.

[55] Senanayake SJ, Gunawardena N, Palihawadana P. Development of the Chronic Kidney Disease Symptom Index – Sri Lanka; a symptom assessment instrument for chronic kidney disease patients. J Postgrad Inst Med. 2017; 4(1): E38 1–12.

[56] Senanayake S, Gunawardena N, Palihawadana P, Bandara P, Haniffa R, Karunarathna R, Kumara P (2017). Symptom burden in chronic kidney disease; a population based cross sectional study. BMC Nephrol.

[57] Craven J, Littlefield C, Rodin G, Murray M (1991). The Endstage renal Disease severity index (ESRD-SI). Psychol Med.

[58] Griffin KW, Friend R, Wadhwa NK (1995). Measuring disease severity in patients with end-stage renal disease: validity of the Craven et al ESRD Severity Index. Psychol Med.

[59] Murphy SP, Powers MJ, Jalowiec A (1985). Psychometric evaluation of the hemodialysis stressor scale. Nurs Res.

[60] Thomas K (2006). Identification of stressors in patients with chronic kidney disease, undergoing long term hemodialysis. Nurs Midwif Res J.

[61] European Medicines Agency. Reflection paper on the regulatory guidance for the use of health-related quality of life (HRQL) measures in the evaluation of medicinal products. 2005. https://www.ema.europa.eu/en/documents/scientific-guideline/reflection-paper-regulatory-guidance-use-healthrelated-quality-life-hrql-measures-evaluation_en.pdf Accessed 22 July 2020.

