# Supplementary material for: Development of a preliminary conceptual model of the patient experience of chronic kidney disease: a targeted literature review and analysis
Source: BMC Nephrol. 2021 Jun 23;22:233. doi: 10.1186/s12882-021-02440-9 (PMC8220773; doi:10.1186/s12882-021-02440-9)
Supplement: Supplementary file 1 — Additional file 1: Table S1 Search terms used for the initial PubMed literature search. Table S2 Predefined inclusion and exclusion criteria for selecting publications for review. Table S3 PRO instrument analysis. Table S4 Signs/symptoms: mentions and prevalence for all publications (N=100). Table S5 Life impacts: mentions and prevalence for all publications (N = 100). Table S6 Signs/symptoms: mentions and prevalence by subpopulation. Table S7 Life impacts: mention and prevalence by subpopulation. Table S8 List of the 138 candidate PRO instruments screened for inclusion in the full analysis Generic HRQOL [file 12882_2021_2440_MOESM1_ESM.docx]

**Development of a preliminary conceptual model of the patient experience of chronic kidney disease: a targeted literature review and analysis**

Jennifer E. Flythe et al.

**Supplementary tables**

**Table S1** Search terms used for the initial PubMed literature search

| **Concept** | **ID** | **Search string** |
| --- | --- | --- |
| **Disease** | #1 | “chronic kidney disease mineral and bone disorder”[MeSH Terms] OR “Chronic renal insufficiency”[MeSH Terms] OR “Dialysis”[MeSH Terms] |
|  | #2 | “chronic kidney disease”[Title/Abstract] OR “CKD”[Title/Abstract] |
|  | #3 | “chronic kidney failure”[Title/Abstract] OR “chronic kidney insufficiency”[Title/Abstract] OR “chronic kidney dysfunction”[Title/Abstract] OR “chronic renal disease”[Title/Abstract] OR “chronic renal failure”[Title/Abstract] OR “chronic renal insufficiency”[Title/Abstract] OR “chronic renal dysfunction”[Title/Abstract] |
|  | #4 | “end-stage renal disease”[Title/Abstract] OR “end stage renal disease”[Title/Abstract] OR “ESRD”[Title/Abstract] OR “end-stage kidney disease”[Title/Abstract] OR “end stage kidney disease”[Title/Abstract] OR “ESKD”[Title/Abstract] |
|  | #5 | “glomerular filtration rate decline”[Title/Abstract] OR “Low eGFR”[Title/Abstract] OR “Estimated Glomerular Filtration Rate Decline”[Title/Abstract] OR “dialysis”[Title/Abstract] |
|  | #6 | “microalbuminuria”[Title/Abstract] OR “macroalbuminuria”[Title/Abstract] OR “Persistent albuminuria Category A1-A3”[Title/Abstract] |
|  | #7 | #1 OR #2 OR #3 OR #4 OR #5 OR #6 |
| **Outcome** | #8 | “quality of life”[MeSH Terms] |
|  | #9 | “patient reported outcome measures”[MeSH Terms] |
|  | #10 | “quality of life”[Title/Abstract] OR “QoL”[Title/Abstract] |
|  | #11 | “Patient Reported Outcomes”[Title/Abstract] OR “patient-reported”[Title/Abstract] OR “patient perspective”[Title/Abstract] OR “PRO”[Title/Abstract] OR “Questionnaire”[Title/Abstract] OR “Clinical Outcomes Assessment”[Title/Abstract] OR “COA tool”[Title/Abstract] |
|  | #12 | “symptom”[Title/Abstract] OR “symptoms”[Title/Abstract] OR “impact”[Title/Abstract] OR “impacts”[Title/Abstract] OR “conceptual model”[Title/Abstract] |
|  | #13 | “health utility”[Title/Abstract] OR “health utilities”[Title/Abstract] OR “disutility”[Title/Abstract] OR “disutilities”[Title/Abstract] |
|  | #14 | “ESRD-SCL-TM”[Title/Abstract] OR “End-Stage Renal Disease Symptom Checklist- Transplantation Module”[Title/Abstract] OR “KDQOL”[Title/Abstract] OR “Kidney Disease Quality of Life instrument”[Title/Abstract] OR “Dialysis Symptom Index”[Title/Abstract] OR “DSI”[Title/Abstract] |
|  | #15 | “EQ-5D”[Title/Abstract] OR EuroQoL[Title/Abstract] OR “SF6”[Title/Abstract] OR “SF12”[Title/Abstract] OR “SF36”[Title/Abstract] OR “short form 6”[Title/Abstract] OR “short form 12”[Title/Abstract] OR “short form 36”[Title/Abstract] OR “HUI”[Title/Abstract] OR “Health utilities index”[Title/Abstract] OR “Patient Health Questionnaire-9”[Title/Abstract] OR “PHQ-9”[Title/Abstract] OR “QUAL-E”[Title/Abstract] OR “Quality of Life at the End of Life Measure”[Title/Abstract] |
|  | #16 | “pain”[Title/Abstract] OR “fatigue”[Title/Abstract] OR “depression”[Title/Abstract] OR “anxiety”[Title/Abstract] |
|  | #17 | “Beck Depression Inventory”[Title/Abstract] OR “BDI”[Title/Abstract] OR “BFI”[Title/Abstract] OR “brief fatigue inventory” OR “Hamilton anxiety scale”[Title/Abstract] OR “HAM-A”[Title/Abstract] OR “HAM-D”[Title/Abstract] OR “FACIT-F”[Title/Abstract] OR “FACIT-Fatigue”[Title/Abstract] OR “Functional Assessment of Chronic Illness Therapy-Fatigue”[Title/Abstract] |
|  | #18 | #8 OR #9 OR #10 OR #11 OR #12 OR #13 OR #14 OR #15 OR #16 OR #17 |
| **Study design** | #19 | “observational study”[Title/Abstract] OR “Interventional study”[Title/Abstract] OR  “RCT”[Title/Abstract] |
|  | #20 | “semi-structured interviews”[Title/Abstract] OR “semi-structured interview”[Title/Abstract] OR “concept elicitation”[Title/Abstract] OR “patient interview”[Title/Abstract] OR “patient interviews”[Title/Abstract] OR “qualitative interview”[Title/Abstract] OR “qualitative interviews”[Title/Abstract] OR “survey”[Title/Abstract] |
|  | #21 | “Systematic review” [Title/Abstract] OR “Systematic Literature review”[Title/Abstract] OR “SLR”[Title/Abstract] OR “Targeted literature review”[Title/Abstract] |
|  | #22 | “study”[Title/Abstract] AND (“longitudinal”[Title/Abstract] OR “observational”[Title/Abstract] OR “cohort”[Title/Abstract] OR “case series”[Title/Abstract] OR “humanistic”[Title/Abstract]) |
|  | #23 | “patients' experiences”[Title/Abstract] OR “patient experience”[Title/Abstract] OR “perceptions of disease”[Title/Abstract] OR “perception of disease”[Title/Abstract] |
|  | #24 | “narrative overview”[Title/Abstract] OR “overview of PROs”[Title/Abstract] OR “PRO review”[Title/Abstract] OR “observational studies”[Title/Abstract] |
|  | #25 | #19 OR #20 OR #21 OR #22 OR #23 OR #24 |
|  | #26 | #7 AND #18 AND #25 |

**Table S2** Predefined inclusion and exclusion criteria for selecting publications for review

|  | Inclusion criteria | Exclusion criteria |
| --- | --- | --- |
| Population of interest | - Patients with CKD | - Patients with other diseases |
| Interventions of interest | - All studies, irrespective of whether or not patients were receiving therapy | - NA |
| Outcomes of interest | - Concepts (i.e. signs/symptoms, and impacts of a disease and its treatments) | - Literature not including an outcome of interest |
| Sources of interest | - Psychometric evaluation of instruments - Patient interview studies - Expert guidelines - Instrument development - Epidemiological studies - Interventional studies - Observational studies - Systematic reviews - Targeted reviews | - Editorials - Case reports |
| Time period  (publication date) | - Articles^a^: 6 November 2009 to   6 November 2019   - Congress abstracts: 1 January 2018 to 6 November 2019 | - Publication date outside the time period |
| Other criteria | - Publications in English | - Non-English-language publications |

^a^For literature searches using the PubMed, EMBASE and Cochrane databases.

CKD, chronic kidney disease; NA, not applicable.

**Table S3** PRO instrument analysis

| **Attribute** | **Description** |
| --- | --- |
| Description | - Brief description of the instrument, focusing on category (e.g. general HRQOL or an anaemia symptoms assessment) |
| *Content* |  |
| Content Validity Strength | - The criteria for the following scores are below   - **Strong**: developed for target indication (kidney diseases), and development process involved patients, literature reviews and clinician experts   - **Medium**: developed for target indication, and development process lacked patient input   - **Weak**: not developed for target indication, or just one type of input involved in the development process |
| Domains | - Whether or not items are grouped by domains |
| Items | - Number of items in the instrument |
| Time to complete | - Time needed for patient to complete the instrument |
| Scoring approach | - Type of response options (e.g. Likert scale, numeric scales) |
| Recall period | - Period of time the patient should be considering when filling out the questionnaire |
| Development process | - Sources and processes used to develop the instrument, specifically literature reviews, concept elicitation interviews from patients, clinician interviews and/or cognitive debriefings with patients - Contributes to strength of content validity |
| Target patient age | - Ages for which the instrument was developed |
| Target patient indication | - Indication for which the instrument was developed - Contributes to strength of content validity |
| Administration mode | - The formats that the instrument is available in (paper, electronic etc.) |
| Translations | - The original language of development and translations |
| Scoring algorithm | - The algorithm used to score the instrument; it may involve different weights being given to distinct domains or concepts; not always available without licence - The availability of the algorithm is a plus |
| *Psychometrics* |  |
| Reference population | - Indication for which the instrument was psychometrically validated |
| Strength of psychometrics | - The scores for psychometric strength were given according to the number of psychometric properties evaluated that met the indicated thresholds   - **Strong**: 4 or 5 properties   - **Medium**: 2 or 3 properties   - **Weak**: 0 or 1 properties |
| Internal consistency reliability | - Usually assessed by Cronbach's alphas to measure the extent to which all the items in a test measure the same concept or construct - Cronbach's alphas should be higher than 0.7 |
| Reproducibility/test–retest reliability | - Determines whether the instrument produces stable scores across a specified time period, as assessed by correlation coefficients between the first and retest scores; the most common assessment is ICC - ICCs should be higher than 0.7 |
| Construct validity | - Assesses construct validity (convergent, divergent/discriminative, known groups) - Generally assessed by correlation coefficients that should range from ≥0.3 to ≥0.6 |
| Minimum clinically important difference | - Level of change deemed to be clinically meaningful (beyond statistical significance) or the smallest change in a treatment outcome that a patient would identify as being important (MCID might have other similar, but necessarily equivalent, measures, like MID, MCD and MCSD) |
| Ability to detect change | - Instrument changes when patients are known to be changing |
| *Other* |  |
| Concept mapping | - Comparison of concepts identified in literature review with concepts covered by the instrument |
| Use in clinical trials | - Whether or not the instrument has been used in clinical trials from October 2014 to October 2019 |
| Use in labelling | - Whether or not the instrument has been used to support labelling claims |

HRQOL, health-related quality of life; ICC, intraclass correlation coefficient; MCD, minimum clinical difference; MCID, minimum clinically important difference; MCSD, minimum clinically significant difference; MID, minimum important difference; PRO, patient-reported outcomes.

**Table S4** Signs/symptoms: mentions and prevalence for all publications (*N* = 100)

| **Signs and symptoms** | **Mentions** | | **Prevalence** | |
| --- | --- | --- | --- | --- |
|  | ***n*** | **%** | ***n*** | **Range (%)** |
| *Pain/discomfort* |  |  |  |  |
| Pain/discomfort^a^ | 57 | 57 | 13 | 10–73 |
| Muscular pain/cramps^a^ | 18 | 18 | 7 | 24–89 |
| Dizziness^a^ | 7 | 7 | 2 | 38–50 |
| Feeling unwell^a^ | 7 | 7 | 2 | 33–55 |
| Headaches^a^ | 6 | 6 | 2 | 31–38 |
| *Energy/fatigue* |  |  |  |  |
| Tiredness/low energy/lethargy/fatigue^a^ | 42 | 42 | 7 | 31–100 |
| Dyspnoea/shortness of breath^a^ | 17 | 17 | 8 | 15–80 |
| Weakness (muscular strength)^a^ | 11 | 11 | 3 | 19–61 |
| *Sleep-related* |  |  |  |  |
| Disturbed sleep/sleep-related^a^ | 28 | 28 | 13 | 14–94 |
| RLS/leg movements during sleep^a^ | 13 | 13 | 5 | 10–65 |
| Drowsiness | 6 | 6 | 5 | 10–82 |
| Snoring | 3 | 3 | 1 | 16–24 |
| *Gastrointestinal-related* |  |  |  |  |
| Appetite loss/anorexia^a^ | 18 | 18 | 5 | 3–67 |
| Nausea^a^ | 18 | 18 | 8 | 6–59 |
| Vomiting^a^ | 8 | 8 | 5 | 3–24 |
| Constipation^a^ | 7 | 7 | 5 | 8–56 |
| Diarrhoea^a^ | 5 | 5 | 4 | 3–64 |
| GI symptoms^a^ | 4 | 4 | 2 | 18–83 |
| *Urinary-related* |  |  |  |  |
| Altered frequency/volume of urination^a^ | 10 | 10 | 3 | 10–78 |
| Sex-related (e.g. lower sex drive, impotency) | 9 | 9 | 1 | 10 |
| *Skin-/hair-/nails-related* |  |  |  |  |
| Itching/skin problems^a^ | 25 | 25 | 9 | 6–89 |
| Hypothermia^a^ | 4 | 4 | 1 | 72 |
| Bruising | 1 | 1 | NR | NR |
| Hair damaged | 1 | 1 | 1 | 28–38 |
| Nail deterioration | 1 | 1 | 1 | 21–52 |
| *Other* |  |  |  |  |
| Numbness/neuropathy^a^ | 12 | 12 | 5 | 14–41 |
| Swelling^a^ | 9 | 9 | 3 | 7–43 |
| Frailty/fracture^a^ | 8 | 8 | 4 | 5–43 |
| Eye-related (retinopathy, lack of vision)^a^ | 6 | 6 | 2 | 14–17 |
| Mouth problems/taste^a^ | 5 | 5 | 4 | 7–83 |
| Thirst^a^ | 5 | 5 | 3 | 19–63 |
| Infections^a^ | 4 | 4 | 2 | 19–56 |
| Pallor | 3 | 3 | 1 | 79 |
| Weight change/loss^a^ | 3 | 3 | 2 | 10–39 |
| Balance disorders^a^ | 2 | 2 | 1 | 44 |
| Diabetes-related complications | 2 | 2 | 1 | 20–100 |
| Cough^a^ | 1 | 1 | 1 | 19 |
| Halitosis | 1 | 1 | NR | NR |
| Olfactory-related^a^ | 1 | 1 | 1 | 22 |
| Palpitations^a^ | 1 | 1 | 1 | 14 |

Table shows number (%) of publications that mention each sign/symptom, number of publications reporting prevalence data and range of prevalence values (%).

^a^Presence in qualitative literature.

GI, gastrointestinal; NR, not reported; RLS, restless legs syndrome.

**Table S5** Life impacts: mentions and prevalence for all publications (*N* = 100)

| **Life impacts** | **Mentions** | | **Prevalence** | |
| --- | --- | --- | --- | --- |
|  | **n** | **%** | **n** | **Range (%)** |
| *Psychological/emotional strain* |  |  |  |  |
| Anxiety/depression^a^ | 40 | 49 | 11 | 5–83 |
| Mental impact^a^ | 26 | 26 | 1 | 13–26 |
| Mood change/irritability^a^ | 12 | 12 | 2 | 24–50 |
| Emotional impact^a^ | 37 | 37 | 2 | 34–82 |
| Vitality^a^ | 23 | 23 | NR | NR |
| Worries^a^ | 12 | 12 | 1 | 10 |
| Frustration^a^ | 10 | 10 | NR | NR |
| Fear^a^ | 8 | 8 | 1 | 10 |
| Impact on self-image^a^ | 6 | 6 | 2 | 5–67 |
| Stress^a^ | 6 | 6 | 1 | 12–22 |
| Uncertainty^a^ | 6 | 6 | NR | NR |
| Denial^a^ | 5 | 5 | 1 | 22 |
| Satisfaction with life^a^ | 4 | 4 | NR | NR |
| *Cognitive impairment* |  |  |  |  |
| Cognitive impairment (memory, concentration, confusion)^a^ | 27 | 27 | 7 | 5–61 |
| Attention and executive functions | 3 | 3 | 1 | 32 |
| *Dietary habit disruption* |  |  |  |  |
| Diet/food changes/related^a^ | 23 | 23 | 4 | 14–83 |
| *Interference with daily living* |  |  |  |  |
| Physical functioning^a^ | 43 | 43 | 5 | 14–83 |
| ADL/daily/regular activities^a^ | 24 | 24 | 5 | 9–80 |
| Mobility problems^a^ | 16 | 16 | 8 | 12–100 |
| Work/ability to work^a^ | 13 | 13 | 3 | 16–55 |
| Self-care issues^a^ | 10 | 10 | 5 | 8–88 |
| Activity impairment^a^ | 9 | 9 | 3 | 28–89 |
| Loss of freedom/independence^a^ | 9 | 9 | NR | NR |
| *Interference with social relationships* |  |  |  |  |
| Social impact^a^ | 34 | 34 | 3 | 5–60 |
| Sex-life impact^a^ | 15 | 15 | 3 | 10–38 |
| *Other* |  |  |  |  |
| General health perception^a^ | 26 | 26 | 3 | 17–36 |
| Financial impact^a^ | 6 | 6 | NR | NR |
| Treatment-related impact^a^ | 5 | 5 | NR | NR |

Table shows number (%) of publications that mention each impact, number of publications reporting prevalence data and range of prevalence values (%).

^a^Presence in qualitative literature.

ADL, activities of daily living; NR, not reported.

**Table S6** Signs/symptoms: mentions and prevalence by subpopulation

| **Signs/symptoms** | **General (*N* = 48)** | | | | **Diabetes (*N* = 11)** | | | | **Anaemia (*N* = 7)** | | | |
| --- | --- | --- | --- | --- | --- | --- | --- | --- | --- | --- | --- | --- |
|  | **Mentions** | | **Prevalence** | | **Mentions** | | **Prevalence** | | **Mentions** | | **Prevalence** | |
|  | **n** | **%** | **n** | **Range (%)** | **n** | **%** | **n** | **Range (%)** | **n** | **%** | **n** | **Range (%)** |
| *Pain/discomfort* |  |  |  |  |  |  |  |  |  |  |  |  |
| Pain/discomfort | 28 | 58 | 6 | 29–75 | 4 | 36 | NR | NR | 5 | 71 | 1 | 45–68 |
| Muscular pain/cramps | 8 | 17 | 4 | 54–89 | 2 | 18 | 1 | 23–44 | – | – | – | – |
| Headaches | 3 | 6 | NR | NR | – | – | – | – | – | – | – | – |
| Feeling unwell | 2 | 4 | NR | NR | 3 | 27 | 1 | 33–52 | – | – | – | – |
| *Energy/fatigue* |  |  |  |  |  |  |  |  |  |  |  |  |
| Tiredness/low energy/lethargy/fatigue | 15 | 31 | 2 | 35–100 | 3 | 27 | NR | NR | 6 | 86 | 1 | 8–50 |
| Dyspnoea/shortness of breath | 6 | 12 | 2 | 34–67 | 1 | 9 | 1 | 18–36 | 1 | 14 | NR | NR |
| Weakness (muscular strength) | 5 | 10 | 2 | 53–61 | 1 | 9 | NR | NR | – | – | – | – |
| *Sleep-related* |  |  |  |  |  |  |  |  |  |  |  |  |
| Disturbed sleep/sleep-related | 16 | 33 | 8 | 22–94 | – | – | – | – | – | – | – | – |
| RLS/leg movements during sleep | 8 | 17 | 2 | 19–26 | – | – | – | – | – | – | – | – |
| Drowsiness | 3 | 6 | 2 | 59 | – | – | – | – | – | – | – | – |
| Snoring | 2 | 4 | NR | NR | – | – | – | – | – | – | – | – |
| *Gastrointestinal-related* |  |  |  |  |  |  |  |  |  |  |  |  |
| Appetite loss/anorexia | 7 | 15 | 2 | 39–67 | 1 | 9 | NR | NR | – | – | – | – |
| Nausea | 4 | 8 | 2 | 22–38 | 2 | 18 | NR | NR | – | – | – | – |
| Constipation | 2 | 4 | 2 | 45–46 | – | – | – | – | – | – | – | – |
| Vomiting | 2 | 4 | 1 | 11 | 1 | 9 | NR | NR | – | – | – | – |
| Diarrhoea | 1 | 2 | 1 | 16 | – | – | – | – | – | – | – | – |
| GI symptoms | 1 | 2 | 1 | 83 | 1 | 9 | 1 | 18–36 | – | – | – | – |
| *Urinary-related* |  |  |  |  |  |  |  |  |  |  |  |  |
| Urinary system-related | 5 | 10 | 1 | 78 | 2 | 18 | NR | NR | – | – | – | – |
| Sex-related (e.g. lower sex drive, impotency) | 5 | 10 | 1 | 71–73 | – | – | – | – | – | – | – | – |
| *Skin-/hair-/nails-related* |  |  |  |  |  |  |  |  |  |  |  |  |
| Itching/skin problems | 8 | 17 | 3 | 21–89 | 2 | 18 | NR | NR | – | – | – | – |
| Hypothermia | 2 | 4 | 1 | 72 | – | – | – | – | – | – | – | – |
| Bruising | 1 | 2 | NR | NR | – | – | – | – | – | – | – | – |
| *Other* |  |  |  |  |  |  |  |  |  |  |  |  |
| Frailty/fracture | 4 | 8 | 2 | 8–43 | 1 | 9 | 1 | 17 | – | – | – | – |
| Mouth problems/taste | 3 | 6 | 2 | 31–83 | – | – | – | – | – | – | – | – |
| Swelling | 3 | 6 | 1 | 30 | 2 | 18 | NR | NR | – | – | – | – |
| Numbness/neuropathy | 2 | 4 | 1 | 15 | 2 | 18 | 1 | 35 | 1 | 14 | NR | NR |
| Weight change/loss | 2 | 4 | 1 | 39 | – | – | – | – | – | – | – | – |
| Balance disorders | 1 | 2 | 1 | 44 | – | – | – | – | – | – | – | – |
| Eye-related (e.g. blurred vision) | 1 | 2 | 1 | 17 | 1 | 9 | NR | NR | 1 | 14 | NR | NR |
| Infections | 1 | 2 | 1 | 56 | 1 | 9 | NR | NR | – | – | – | – |
| Pallor | 1 | 2 | NR | NR | 1 | 9 | NR | NR | – | – | – | – |
| Smell alteration | 1 | 2 | 1 | 22 | – | – | – | – | – | – | – | – |
| Thirst | 1 | 2 | NR | NR | – | – | – | – | – | – | – | – |
| Diabetes-related complications | – | – | – | – | 2 | 18 | 1 | 20–100 | 1 | 14 | NR | NR |

**Table S6** continued

| **Signs/symptoms** | **CKD stages 1–3 (*N* = 9)** | | | | **CKD stages 4–5 (*N* = 14)** | | | | **Dialysis (*N* = 26)** | | | |
| --- | --- | --- | --- | --- | --- | --- | --- | --- | --- | --- | --- | --- |
|  | **Mentions** | | **Prevalence** | | **Mentions** | | **Prevalence** | | **Mentions** | | **Prevalence** | |
|  | **n** | **%** | **n** | **Range (%)** | **n** | **%** | **n** | **Range (%)** | **n** | **%** | **n** | **Range (%)** |
| *Pain/discomfort* |  |  |  |  |  |  |  |  |  |  |  |  |
| Pain/discomfort | 4 | 44 | 2 | 38–60 | 8 | 57 | 4 | 58–100 | 19 | 73 | 5 | 10–75 |
| Muscular pain/cramps | 1 | 11 | 1 | 55 | 1 | 7 | 1 | 83 | 8 | 31 | 2 | 24–69 |
| Dizziness | – | – | – | – | – | – | – | – | 7 | 27 | 2 | 38–50 |
| Headaches | – | – | – | – | – | – | – | – | 3 | 11 | 2 | 31–38 |
| Feeling unwell | 1 | 11 | NR | NR | 1 | 7 | NR | NR | 2 | 8 | 1 | 36 |
| *Energy/fatigue* |  |  |  |  |  |  |  |  |  |  |  |  |
| Tiredness/low energy/lethargy/fatigue | 2 | 22 | 1 | 8–24 | 5 | 36 | 2 | 17–76 | 16 | 62 | 4 | 14–71 |
| Dyspnoea/shortness of breath | – | – | – | – | 3 | 21 | 2 | 59–80 | 8 | 31 | 4 | 21–66 |
| Weakness (muscular strength) | – | – | – | – | 2 | 14 | NR | NR | 4 | 15 | 1 | 19 |
| *Sleep-related* |  |  |  |  |  |  |  |  |  |  |  |  |
| Disturbed sleep/sleep-related | 1 | 11 | 1 | 55 | 4 | 29 | 3 | 14–66 | 10 | 38 | 4 | 24–80 |
| RLS/leg movements during sleep | – | – | – | – | 2 | 14 | 1 | 65 | 5 | 19 | 3 | 10–33 |
| Drowsiness | – | – | – | – | 2 | 14 | 2 | 52–82 | 2 | 8 | 2 | 36–64 |
| Snoring | – | – | – | – | 1 | 7 | 1 | 16–24 | – | – | – | – |
| *Gastrointetinal-related* |  |  |  |  |  |  |  |  |  |  |  |  |
| Nausea | – | – | – | – | 3 | 21 | 3 | 19–59 | 10 | 38 | 4 | 14–42 |
| Appetite loss/anorexia | – | – | – | – | 3 | 21 | 1 | 32 | 9 | 35 | 3 | 29–53 |
| Constipation | – | – | – | – | 1 | 7 | 1 | 48 | 5 | 19 | 3 | 15–56 |
| Diarrhoea | – | – | – | – | 1 | 7 | 1 | 42 | 4 | 15 | 3 | 9–64 |
| Vomiting | – | – | – | – | 2 | 14 | 2 | 6–23 | 4 | 15 | 3 | 19–24 |
| GI symptoms (general) | 1 | 11 | NR | NR | 1 | 7 | NR | NR | 1 | 4 | NR | NR |
| *Urinary-related* |  |  |  |  |  |  |  |  |  |  |  |  |
| Sex-related (e.g. lower sex drive, impotency) | – | – | – | – | 1 | 7 | NR | NR | 2 | 8 | 1 | 10 |
| Urinary-system-related | 1 | 11 | NR | NR | 2 | 14 | 1 | 38–45 | 1 | 4 | 1 | 10–19 |
| *Skin-/hair-/nails-related* |  |  |  |  |  |  |  |  |  |  |  |  |
| Itching/skin problems | 2 | 22 | NR | NR | 4 | 29 | 2 | 32–84 | 11 | 42 | 5 | 6–83 |
| Hair damaged | – | – | – | – | – | – | – | – | 1 | 4 | 1 | 28–39 |
| Hypothermia | – | – | – | – | 1 | 7 | NR | NR | 1 | 4 | NR | NR |
| Nail deterioration | – | – | – | – | – | – | – | – | 1 | 4 | 1 | 21–52 |
| *Other* |  |  |  |  |  |  |  |  |  |  |  |  |
| Numbness/neuropathy | 1 | 11 | NR | NR | 1 | 7 | NR | NR | 6 | 23 | 3 | 14–41 |
| Swelling | – | – | – | – | 1 | 7 | NR | NR | 3 | 12 | 2 | 7–43 |
| Thirst | – | – | – | – | 1 | 7 | 1 | 63 | 3 | 12 | 2 | 19–62 |
| Eye-related | 2 | 22 | NR | NR | 1 | 7 | NR | NR | 2 | 8 | 1 | 14 |
| Balance disorders | – | – | – | – | – | – | – | – | 1 | 4 | NR | NR |
| Cough | – | – | – | – | – | – | – | – | 1 | 4 | 1 | 19 |
| Frailty/fracture | 2 | 22 | 1 | 5–15 | – | – | – | – | 1 | 4 | NR | NR |
| Infections | 1 | 11 | NR | NR | 1 | 7 | NR | NR | 1 | 4 | 1 | 19–31 |
| Mouth problems/taste | – | – | – | – | 2 | 14 | 2 | 7–33 | 1 | 4 | 1 | 36 |
| Pallor | – | – | – | – | – | – | – | – | 1 | 4 | 1 | 79 |
| Palpitations | – | – | – | – | – | – | – | – | 1 | 4 | 1 | 14 |
| Weight change/loss | – | – | – | – | – | – | – | – | 1 | 4 | 1 | 10 |
| Halitosis | – | – | – | – | 1 | 7 | NR | NR | – | – | – | – |

Table shows number (%) of publications that mention each sign/symptom, number of publications reporting prevalence data and range of prevalence values (%) for each subpopulation.

– , not mentioned; CKD, chronic kidney disease; GI, gastrointestinal; NR, not reported; RLS, restless legs syndrome.

**Table S7** Life impacts: mentions and prevalence by subpopulation

| **Life impacts** | **General (*N* = 48)** | | | | **Diabetes (*N* = 11)** | | | | **Anaemia (*N* = 7)** | | | |
| --- | --- | --- | --- | --- | --- | --- | --- | --- | --- | --- | --- | --- |
|  | **Mentions** | | **Prevalence** | | **Mentions** | | **Prevalence** | | **Mentions** | | **Prevalence** | |
|  | **n** | **%** | **n** | **Range (%)** | **n** | **%** | **n** | **Range (%)** | **n** | **%** | **n** | **Range (%)** |
| *Psychological/emotional strain* |  |  |  |  |  |  |  |  |  |  |  |  |
| Anxiety/depression | 27 | 56 | 9 | 6–39 | 3 | 27 | 2 | 6–83 | 1 | 14 | NR | NR |
| Mental impact | 13 | 27 | 1 | 20–21 | 6 | 55 | 1 | 13–26 | 3 | 43 | NR | NR |
| Mood change/irritability | 5 | 10 | 1 | 50 | 4 | 36 | NR | NR | – | – | – | – |
| Emotional impact | 17 | 35 | 2 | 34–82 | 5 | 45 | NR | NR | 3 | 43 | NR | NR |
| Vitality | 9 | 19 | 1 | 20–24 | 4 | 36 | NR | NR | 6 | 86 | 1 | 50–60 |
| Fear | 4 | 8 | NR | NR | 1 | 9 | NR | NR | – | – | – | – |
| Frustration | 4 | 8 | NR | NR | 1 | 9 | NR | NR | – | – | – | – |
| Uncertainty | 4 | 8 | NR | NR | – | – | – | – | – | – | – | – |
| Worries | 4 | 8 | NR | NR | 1 | 9 | NR | NR | – | – | – | – |
| Impact on self-image | 3 | 6 | 1 | 67 | – | – | – | – | – | – | – | – |
| Satisfaction with life | 3 | 6 | NR | NR | – | – | – | – | – | – | – | – |
| Stress | 3 | 6 | 1 | 12–22 | – | – | – | – | – | – | – | – |
| Denial | 1 | 2 | 1 | 22 | 1 | 9 | NR | NR | – | – | – | – |
| *Cognitive impairment* |  |  |  |  |  |  |  |  |  |  |  |  |
| Cognitive impairment (memory, concentration, confusion) | 15 | 31 | 4 | 6–71 | 1 | 9 | 1 | 36 | – | – | – | – |
| Attention and executive functions | 1 | 2 | 1 | 32 | – | – | – | – | – | – | – | – |
| *Dietary habit disruption* |  |  |  |  |  |  |  |  |  |  |  |  |
| Diet/food changes/related | 11 | 23 | 2 | 35–74 | 4 | 36 | 1 | 37–43 | – | – | – | – |
| *Interference with daily living* |  |  |  |  |  |  |  |  |  |  |  |  |
| Physical functioning | 25 | 52 | 4 | 22–83 | 6 | 55 | NR | NR | 6 | 86 | NR | NR |
| ADL/daily/regular activities | 12 | 25 | 2 | 9–46 | – | – | – | – | 2 | 28 | 1 | 17–83 |
| Mobility problems | 10 | 21 | 3 | 23–61 | 1 | 9 | NR | NR | 1 | 14 | NR | NR |
| Work/ability to work | 8 | 17 | 2 | 16–55 | – | – | – | – | – | – | – | – |
| Self-care issues | 4 | 8 | NR | NR | 1 | 9 | NR | NR | 2 | 28 | NR | NR |
| Activity impairment | 3 | 6 | 1 | 89 | 2 | 18 | NR | NR | 3 | 43 | 2 | 8–22 |
| Loss of freedom/independence | 3 | 6 | NR | NR | 1 | 9 | NR | NR | 1 | 14 | 1 | 8–22 |
| *Interference with social relationships* |  |  |  |  |  |  |  |  |  |  |  |  |
| Social impact | 15 | 31 | 4 | 12–89 | 7 | 64 | NR | NR | 4 | 57 | 1 | 76–83 |
| Sex-life impact | 7 | 15 | 1 | 33 | – | – | – | – | 1 | 14 | 1 | 71–73 |
| *Other* |  |  |  |  |  |  |  |  |  |  |  |  |
| General health perception | 13 | 27 | 1 | 17–19 | 3 | 27 | NR | NR | 4 | 57 | NR | NR |
| Financial impact | 2 | 4 | NR | NR | 2 | 18 | NR | NR | 1 | 14 | NR | NR |
| Treatment-related impact | 2 | 4 | NR | NR | 1 | 9 | NR | NR | – | – | – | – |

**Table S7** continued

| **Life impacts** | **CKD stages 1–3 (*N* = 9)** | | | | **CKD stages 4–5 (*N* = 14)** | | | | **Dialysis (*N* = 26)** | | | |
| --- | --- | --- | --- | --- | --- | --- | --- | --- | --- | --- | --- | --- |
|  | **Mentions** | | **Prevalence** | | **Mentions** | | **Prevalence** | | **Mentions** | | **Prevalence** | |
|  | **n** | **%** | **n** | **Range (%)** | **n** | **%** | **n** | **Range (%)** | **n** | **%** | **n** | **Range (%)** |
| *Psychological/emotional strain* |  |  |  |  |  |  |  |  |  |  |  |  |
| Anxiety/depression | 5 | 55 | 4 | 5–33 | 6 | 43 | 4 | 12–65 | 14 | 54 | 6 | 5–57 |
| Emotional impact | – | – | – | – | 2 | 14 | NR | NR | 14 | 54 | 1 | 45 |
| Vitality | 2 | 22 | NR | NR | 2 | 14 | NR | NR | 6 | 23 | NR | NR |
| Worries | – | – | – | – | 1 | 7 | NR | NR | 5 | 19 | 1 | 10 |
| Frustration | – | – | – | – | – | – | – | – | 5 | 19 | NR | NR |
| Fear | – | – | – | – | – | – | – | – | 3 | 12 | 1 | 10 |
| Impact on self-image | – | – | – | – | – | – | – | – | 3 | 12 | 1 | 5 |
| Stress | – | – | – | – | – | – | – | – | 3 | 12 | NR | NR |
| Denial | – | – | – | – | – | – | – | – | 3 | 12 | NR | NR |
| Mood change/irritability | 1 | 11 | NR | NR | 2 | 14 | NR | NR | 2 | 8 | 1 | 24 |
| Satisfaction with life | – | – | – | – | – | – | – | – | 1 | 4 | NR | NR |
| Uncertainty | – | – | – | – | 1 | 7 | NR | NR | 1 | 4 | NR | NR |
| *Cognitive impairment* |  |  |  |  |  |  |  |  |  |  |  |  |
| Cognitive impairment (memory, concentration, confusion) | 3 | 33 | 1 | 7–14 | 2 | 14 | NR | NR | 9 | 35 | 3 | 5–52 |
| Mental impact | – | – | – | – | 1 | 7 | NR | NR | 6 | 23 | NR | NR |
| Attention and executive functions | 1 | 11 | NR | NR | 1 | 7 | NR | NR | 1 | 4 | NR | NR |
| *Dietary habit disruption* |  |  |  |  |  |  |  |  |  |  |  |  |
| Diet/food changes/related | – | – | – |  | 1 | 7 | 1 | 40 | 7 | 27 | 1 | 43–48 |
| *Interference with daily living* |  |  |  |  |  |  |  |  |  |  |  |  |
| ADL/daily/regular activities | 3 | 33 | 3 | 13–49 | 2 | 14 | 2 | 53–80 | 10 | 38 | 2 | 29–60 |
| Physical functioning | 2 | 22 | 1 | 22–61 | 2 | 14 | NR | NR | 9 | 35 | 1 | 14 |
| Mobility problems | 3 | 33 | 3 | 14–45 | 3 | 21 | 3 | 46–100 | 5 | 19 | 3 | 14–70 |
| Work/ability to work | 1 | 11 | 1 | 16–55 | – | – | – | – | 5 | 19 | 1 | 19 |
| Loss of freedom/independence | – | – | – | – | – | – | – | – | 4 | 15 | NR | NR |
| Activity impairment | 1 | 11 | 1 | 37–52 | 1 | 7 | 1 | 48 | 3 | 12 | 2 | 29–52 |
| Self-care issues | 2 | 22 | 2 | 8–18 | 3 | 21 | 3 | 20–68 | 3 | 12 | 3 | 19–88 |
| *Interference with social relationships* |  |  |  |  |  |  |  |  |  |  |  |  |
| Social impact | 1 | 11 | 1 | 12–26 | 1 | 7 | NR | NR | 10 | 38 | 2 | 5–28 |
| Sex-life impact | – | – | – | – | 1 | 7 | NR | NR | 6 | 23 | 1 | 10 |
| *Other* |  |  |  |  |  |  |  |  |  |  |  |  |
| General health perception | 1 | 11 | NR | NR | 1 | 7 | NR | NR | 8 | 31 | 2 | 17–36 |
| Financial impact | – | – | – | – | – | – | – | – | 2 | 8 | NR | NR |
| Treatment-related impact | – | – | – | – | – | – | – | – | 2 | 8 | NR | NR |

Table shows number (%) of publications that mention each impact, number of publications reporting prevalence data and range of prevalence values (%) for each subpopulation.

– , not mentioned; ADL, activities of daily living; CKD, chronic kidney disease; NR, not reported.

**Table S8** List of the 138 candidate PRO instruments screened for inclusion in the full analysis

**Generic HRQOL**

| **Name** | **Acronym** | **Selected** |
| --- | --- | --- |
| 36-Item Short-Form Survey | SF-36, SF-36 VS, RAND36, VR-36 | YES |
| EuroQoL – five dimension | EQ-5D, EQ-5D-5L, EQ-5D-3L, EQ-VAS, ED-5D | YES |
| SF12 Health Survey | SF-12, SF-12 V.2 | NO |
| Memorial Symptom Assessment Scale | MSAS, MSAS-GDI, MSAS-SF | NO |
| Edmonton Symptom Assessment System Revised | ESASr, ESAS | NO |
| World Health Organization Quality of Life Brief Scale | WHOQOL-BREF, WHOQOL-100, WHO-5 | NO |
| Sickness Impact Profile | SIP | NO |
| Symptom Distress Scale | SDS, PSDS | NO |
| Ferrans and Powers Quality of Life Index | QLI | NO |
| Health Utilities Index | HUI | NO |
| Ines Perception Questionnaire | IPQ, IPQ-R, B-IPQ | NO |
| Nottingham Health Profile | NHP | NO |
| Quality of Well-Being Scale | QWB, QWB-SA | NO |
| Satisfaction with Life Scale | SWLS | NO |
| Illness Effects Questionnaire | IEQ | NO |
| Hemodialysis Quality of Life Questionnaire | HQL | NO |
| Illness Intrusiveness Rating Scale | IIRS | NO |
| Australian Modified Karnofsky Performance status | AKPS | NO |
| McGill Quality-of-Life Questionnaire | MQOL | NO |
| Short-Form Six-Dimension | SF-6D | NO |
| Patient-Reported Outcomes Measurement Information System 57/43/29 Question Short Form | PROMIS-57/43/29, PROMIS | NO |
| Quality of Life at the End of Life | QUAL-E | NO |
| 20-Item Short-Form Survey Instrument | SF-20 | NO |
| Acceptance of Illness Scale | AIS | NO |
| Affect Balance Scale | ABS | NO |
| Basic Activities of Daily Living | BADL | NO |
| Implicit Models of Illness Questionnaire | IMIQ | NO |
| International Support Evaluation List-16 Item | ISEL-16 | NO |
| Life Satisfaction Index | LSI | NO |
| Physical Functioning Questionnaire | PFQ-Adult | NO |
| Psychological Adjustment to Illness Scale | PAIS | NO |
| Revised Hopkins Symptom Checklist | SCL90-R, RHSC | NO |
| Rotterdam Symptom Checklist | RSCL | NO |
| Social Difficulties Inventory | SDI | NO |
| Strategies Used by People to Promote Health | SUPPH | NO |
| Patient Global Impression | PGIC, PGIC-S | NO |
| Self-Efficacy for Managing Chronic Disease Scale | SEMCD | NO |
| Self-Management: Partners in Health Scale | SMPIH | NO |
| Barthel Activities of Daily Living Index | – | NO |

**Generic concept-specific**

| **Name** | **Acronym** | **Selected** |
| --- | --- | --- |
| *Depression* |  |  |
| Beck Depression Inventory | BDI, BDI-II | YES |
| Hospital Anxiety and Depression Scale | HADS | YES |
| Patient Health Questionnaire | PHQ, PHQ-9 | YES |
| Centre for Epidemiologic Studies Depression Scale | CES-D, CES-D-4, CES-D-10 | NO |
| Cognitive Depression Index | CDI | NO |
| Geriatric Depression Scale-15 Item | GDS-15 | NO |
| Major Depression Inventory | MDI | NO |
| 16-Item Quick Inventory of Depressive Symptomatology | QIDS | NO |
| *Cognitive function* |  |  |
| Mini-Mental State Examination | MMSE, MMSE 3MS | NO |
| Trail Making Test | TMT | YES |
| Clock Drawing Test | CDT | NO |
| Digit Symbol Substitution Test | DSST | NO |
| Raven’s Progressive Matrices | RPM | NO |
| Rey–Osterreith Complex Figure | ROCF | NO |
| Stroop Test | – | NO |
| Verbal Fluency Test | NA | NO |
| Wechsler Adult Intelligence Scale | WAIS | NO |
| Wisconsin Card Sorting Test | WCST | NO |
| *Physical activity* |  |  |
| 6-Minute Walk Test | 6MWT | NO |
| Human Activity Profile | HAP | YES |
| 2-Minute Walk Test | – | NO |
| 6-Metre Walking Speed | – | NO |
| 24-Hour Body Activity Measure | – | NO |
| Duke Activity Status Index-Physical | DASI-Physical, DASI | NO |
| General Practice Physical Activity Questionnaire | GPPAQ | NO |
| International Physical Activity Questionnaire | IPAQ, IPAQ-SF | NO |
| Lower Extremity Functional Scale | LEFS | NO |
| Physical Activity Scale for the Elderly | PASE | NO |
| Self-Efficacy for Exercise | SEE | NO |
| Short Physical Performance Battery | SPPB | NO |
| Sit-to-Stand Test | STST | NO |
| Timed Up and Go Test | TUG test | NO |
| *Pain* |  |  |
| Brief Pain Inventory | BPI | YES |
| Average Daily Pain Scores | ADP | NO |
| Wong–Baker FACES Pain Rating Scale | WBFPRS | NO |
| *Sleep* |  |  |
| Pittsburgh Sleep Quality Index | PSQI | YES |
| 24-Hour Sleep Quality | – | NO |
| Epworth Sleepiness Scale | ESS, JESS | NO |
| Functional Outcomes of Sleep Questionnaire | FOSQ | NO |
| Insomnia Severity Index | ISI | NO |
| Medical Outcomes Study Sleep Scale | MOS-SS | NO |
| Sleep Heart Health Study Sleep Habits Questionnaire | SHHS-Sleep habit | NO |
| *Fatigue* |  |  |
| Functional Assessment of Chronic Illness Therapy-Fatigue Scale | FACIT-F, FACIT-fatigue | YES |
| Fatigue Severity Scale | FSS | NO |
| Functional Assessment of Cancer Therapy-Fatigue | FACT-F | NO |
| Multidimensional Fatigue Inventory | MFI | NO |

**Generic concept-specific (cont.)**

| **Name** | **Acronym** | **Selected** |
| --- | --- | --- |
| *Itching* |  |  |
| Skindex (pruritus-specific quality of life) | QoL-Skindex-10 | YES |
| 5-D Itch Scale | – | NO |
| Pruritus-specific sleep quality | Itch MOS | NO |
| Worst Itching Intensity Numerical Rating Scale | – | NO |
| *Restless legs syndrome* |  |  |
| Restless Legs Syndrome-6 Scale | RLS-6 | YES |
| Cambridge-Hopkins diagnostic questionnaire for Restless Legs Syndrome | CH-RLSq | NO |
| International Restless Legs Syndrome Study Group Severity Scale | IRLSSGSS | NO |
| RLS Questionnaire | RLS-Q | NO |
| *Coping behaviours* |  |  |
| Coping Strategy Indicator | CSI, CSI-SF | YES |
| Brief - Coping Orientation to Problems Experienced | Brief - COPE | NO |
| Jalowiec Coping Scale | JCS | NO |

**Generic concept-specific (cont.)**

| **Name** | **Acronym** | **Concept** | **Selected** |
| --- | --- | --- | --- |
| *Other concepts* |  |  |  |
| Functional Assessment of Anorexia/Cachexia Therapy | FAACT | Anorexia/cachexia | YES |
| Functional Assessment of Cancer Therapy-Anemia | FACT-An | Anaemia | YES |
| Hand Grip Strength Test | HGS | Strength | YES |
| Work Productivity and Activity Impairment | WPAI, WPAI: ANS | Work productivity | YES |
| Cohen's Perceived Stress Scale | PSS-4 | Stress | NO |
| Columbia-Suicide Severity Rating Scale | C-SSRS | Suicide | NO |
| Edmonton Frail Scale | EFS | Frailty | NO |
| Falls Efficacy Scale International | FES-I | Fear (anxiety) | NO |
| Fluid Management Survey | – | Fluid management | NO |
| Gastrointestinal Rating Scale | GSRS, GSRS-self | GI symptoms | NO |
| Gastrointestinal Quality of Life Index | GIQLI | GI symptoms | NO |
| Generalized Anxiety Disorder 7 Scale | GAD-7 | Anxiety | NO |
| Instrumental Activities of Daily Living | IADL | ADL | NO |
| Judgment of Line Orientation | JLO | Visual skills | NO |
| Lifestyle Defense Mechanism Inventory | LDMI | Emotional | NO |
| Multidimensional Scale of Perceived Social Support | MSPSS | Social support | NO |
| Rationality/Emotional Defensiveness Scale | R/ED scale | Emotional | NO |
| Thirst Distress Scale | TDS | Thirst | NO |

ADL, activities of daily living; GI, gastrointestinal.

**Kidney disease-specific HRQOL**

| **Name** | **Acronym** | **Selected** |
| --- | --- | --- |
| CHOICE Health Experience Questionnaire | CHEQ | YES |
| Chronic Kidney Disease Quality of Life | CKD QOL | YES |
| Chronic Kidney Disease-Symptom Burden Index | CKD-SBI | YES |
| Dialysis Symptom Index | DSI, DSI-30 | YES |
| Kidney Disease Behavior Inventory | KDBI | YES |
| Kidney Disease Quality of Life | KDQOL, KDQ | YES |
| Kidney Disease Quality of Life-36 | KDQOL-36, KDQOL-KSS | YES |
| Kidney Disease Quality of Life Short Form | KDQOL-SFTM, KDQOL-SF | YES |
| Palliative Care Outcome Scale-Symptoms (Renal) | POS-S Renal | YES |
| Quality of Life Index Dialysis Version | QLI-D | YES |
| World Health Organization Quality of Life Brief Scale in Dialysis | WHOQOL-BREF Dial. | YES |
| Autosomal Dominant Polycystic Kidney Disease Impact Scale | ADPKD-IS, ADPKD-UIS | NO |
| National Kidney Dialysis and Kidney Transplant Study symptom checklist | NKDKTS | NO |
| Perceived Kidney Disease Self-Management Scale | PKDSMS | NO |
| Renal Dependent Individualized Quality of Life Questionnaire | RDI-QLQ, RDQOL | NO |
| Renal Quality of Life Profile | RQLP | NO |
| Symptom Monitoring on Renal Replacement Therapy-Hemodialysis | SMaRRT-HD | NO |

**Kidney disease concept-specific**

| **Name** | **Acronym** | **Concept** | **Selected** |
| --- | --- | --- | --- |
| End-Stage Renal Disease Severity Index | ESRD-SI | Disease severity | YES |
| Focal Segmental Glomerulosclerosis Symptom Impact Questionnaire | FSGS | FSGS symptoms | YES |
| Hemodialysis Fatigue Scale | FAS | Fatigue | YES |
| Hemodialysis Stressor Scale | HSS | Stress | YES |
| CKD-Anaemia Symptoms Questionnaire (AQ) | CKD-AQ | Anaemia | NO |
| End-Stage Renal Disease-Adherence Questionnaire | ESRD-AQ | Treatment adherence | NO |
| 25-Item Chronic Kidney Disease Self-Efficacy Instrument | CKD-SE | Self-efficacy | NO |
